# Supplementary material for: The Efficacy of Short-Term Weight Loss Programs and Consumption of Natural Probiotic Bryndza Cheese on Gut Microbiota Composition in Women
Source: Nutrients. 2021 May 21;13(6):1753. doi: 10.3390/nu13061753 (PMC8224276; doi:10.3390/nu13061753)
Supplement: Supplementary file 1 [file nutrients-13-01753-s001.zip › Nov8 prieƒinok/Supplement 1.pdf]

NUTRIČNÝ PLÁN

KLIENT

Fit Curves .

NÁZOV PLÁNU

Diéta po transplante 1.-3. mesiac

VSTUPNÁ ENERGIA

1500 kcal / 6280.2 KJ

TRVANIE PLÁNU

24.04.2019 - 23.05.2019

NUTRIČNÝ PLÁN ZOSTAVIL

doc. RNDr. Viktor Bielik, PhD.

Popis plánu

Milé dámy FitCurves

Nastavili sme vám 4 hlavné jedlá za deň.

· Pokúste sa mať **mäso maximálne 3-4 x týždenne**.

· **Po večernom aeróbnom tréningu** (alebo 1 hod rýchla chôdza - 5km /h) sa snažte nič nejesť, **len piť čistú vodu**

· Ak budete jedlo pripravovať/variť, môžete ho konzumovať opakovane (**napr. na večeru, čo ostalo z obeda, alebo raňajkovú nátierku 2 dní po sebe a pod.)**

· Miesto olovrantu, nie však skôr ako **3 hodiny** po obede si dajte porciu ovocia. Maximálne však 300g.

. K hlavnému jedlu si vždy môžete pridať zeleninu (napr. rajčiny, papriku, uhorku, poľný šalát)

· **Jedlá striedajte** .

· **Nezmenšujte si veľkosť porcií.**

Ak sa nebudete hýbať, nebude to fungovať :)

Prehľad rozloženia makronutrientov a energie počas dňa

ROZLOŽENIE ENERGIE POČAS DŇA

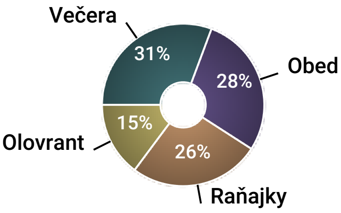

MAKRONUTRIENTY - RAŇAJKY

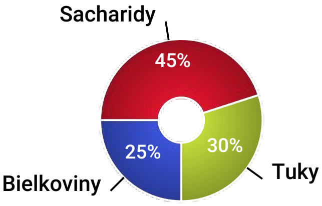

MAKRONUTRIENTY - OBED

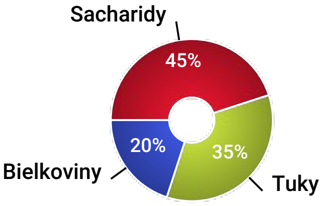

MAKRONUTRIENTY - OLOVRANT

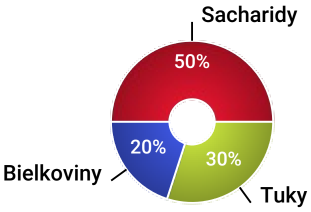

MAKRONUTRIENTY - VEČERA

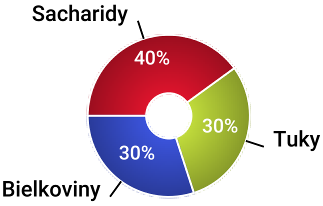

Vysvetlivky k nutričnému plánu

POSTUP A PRIBLIŽNÝ ČAS PRÍPRAVY

S kuchármi a lekármi sme pripravili postup ako jedlá pripraviť chutne a zdravo. Postup prípravy má slúžiť ako odporúčanie a nemusí sa striktne dodržiavať. "Ďalšie ingrediencie" sú väčšinou ingrediencie s veľmi nízkym obsahom energie, ktoré slúžia na dochutenie.

10 min

Postup prípravy:

Ovocie si nakrájajte do misky a na desiatu skonzumujte.

Ďalšie ingrediencie:

Podľa chuti pokvapkajte citrónovou šťavou

ZOZNAM INGREDIENCIÍ, KTORÉ DANÉ JEDLO OBSAHUJE

Pri každej ingrediencii je zobrazené jej množstvo v kuchynských mierach (pre jednoduchosť použitia) a následne je v zátvorke uvedené presné množstvo ingrediencie v gramoch (nejedná sa teda o hmotnosť kuchynskej miery, ale o presné odporúčané množstvo príslušnej ingrediencie).

INGREDIENCIE

¾ x balenie Ingrediencia 1 (127.5 g)

2 x ks Ingrediencia 2 (150 g)

2 a ½ x krajec Ingrediencia 3 (125 g)

4 x ks Ingrediencia 4 (12 g)

ROZLOŽENIE ENERGIE VO VARIANTE JEDLA

Graf znázorňuje množstvo energie, ktorú jednotlivé ingrediencie v jedle obsahujú. Slúži na rozpoznanie ingrediencií s vysokým obsahom energie.

ROZLOŽENIE ENERGIE

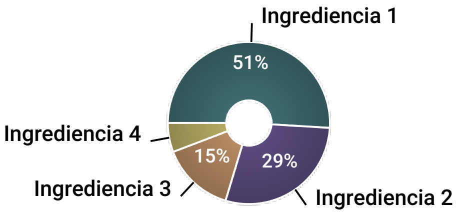

ĎALŠIE INFORMÁCIE O JEDLE (INFORMATÍVNE ZOBRAZENIE, PRIBLIŽNÝCH HODNÔT PRE DANÝ VARIANT)

GL - Glykemická nálož

22 Vysoká GL

Chol. - Cholesterol v miligramoch (mg)

6.2 Chol

Raňajky

Bageta

7 min

Postup prípravy:

Bagetu rozkrojte, potrite maslom a horčicou/kečupom. Obložte vákuovo balenou šunkou a plátkom eidamu a konzumujte spolu s vybranou zeleninou. Dobrú chuť.

Ďalšie ingrediencie:

zelenina (ak na prísady nie ste alergická)

ENERGIA	SACHARIDY	TUKY	BIELKOVINY
375.16 kcal	41.52 g	12.28 g	22.12 g
Ideál: 371.93 kcal	Ideál: 40.82 g	Ideál: 12 g	Ideál: 22.68 g

INGREDIENCIE

½ x kus bageta celozrnná (60 g)
3 x plátok priemer 14.5 cm morčacia šunka (54 g)
1 a ½ x ČL živočíšne maslo (7.5 g)
1 x plátok (9×9 cm) syr eidam, 30% t. v s. neúdený (22 g)
1 a ½ ČL kečup (13.5 g)

ROZLOŽENIE ENERGIE

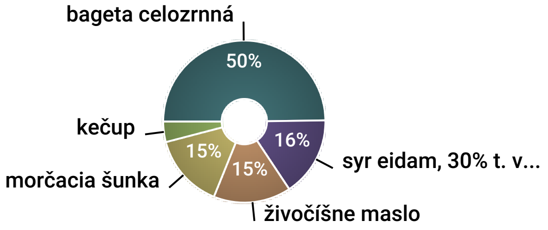

ĎALŠIE INFORMÁCIE O JEDLE

35 Chol

Tofu nátierka s pečivom

10 min

Postup prípravy:

Tofu (pasterizované alebo povarené) rozmačkajte vidličkou a pomiešajte spolu so syrom a horčicou (prípadne aj olejom, ak je uvedený v recepte). Zeleninu poriadne umyte, prípadne ošúpte a nakrájajte na menšie kúsky. Nátierku z tofu naneste na pečivo a na vrch poukladajte plátky zeleniny. Dobrú chuť.

Ďalšie ingrediencie:

nasekaná čerstvá bazalka alebo petržlenová vňať (podľa chuti) - min. 1 lyžicu, zeleninová soľ, korenie, medvedí cesnak (ak na prísady nie ste alergická)

ENERGIA	SACHARIDY	TUKY	BIELKOVINY
392.07 kcal	42.96 g	12.54 g	24.23 g
Ideál: 391.5 kcal	Ideál: 42.97 g	Ideál: 12.63 g	Ideál: 23.87 g

INGREDIENCIE

½ x balenie tofu biele napr. Alfa Bio (90 g)
1 a ½ x krajec chlieb pšeničný biely (75 g)
5 ks cherry paradajky (60 g)
3 ČL mäkký nezrejúci syr napr. Lučina (18 g)
1 ČL horčica plnotučná napr. Snico (9 g)

ROZLOŽENIE ENERGIE

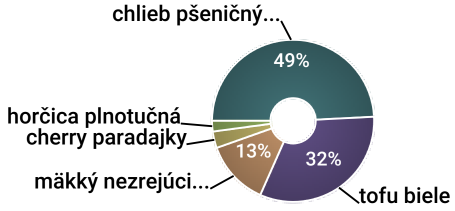

ĎALŠIE INFORMÁCIE O JEDLE

Omeleta vegetarián

10 min

Postup prípravy:

Na panvici orestujte na kocky nakrájaný tofu syr alebo hlivu ústricovú. Po 2-3 min. pridajte špenátové listy, zakryjte pokrievkou a 2-3 min poduste. Potom pridajte vajcia, osoľte štipkou soli, opäť prikryte pokrievkou a duste, kým nie sú vajcia hotové. Zeleninu poriadne umyte a konzumujte spolu s omeletou a pečivom. Dobrú chuť.

ENERGIA	SACHARIDY	TUKY	BIELKOVINY
396.85 kcal	43.21 g	13.74 g	22.42 g
Ideál: 391.5 kcal	Ideál: 42.97 g	Ideál: 12.63 g	Ideál: 23.87 g

INGREDIENCIE

70 g hliva ustricová
2 ks slepačie vajce (110 g)
35 g baby špenát
4 ks cherry paradajky (48 g)
1 a ½ x krajec chlieb celozrnný ražný (ideálne kváskový) (75 g)

ROZLOŽENIE ENERGIE

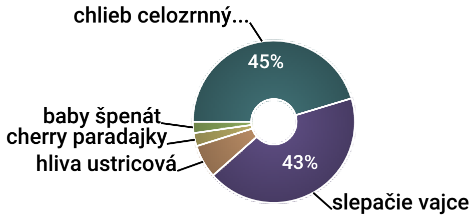

ĎALŠIE INFORMÁCIE O JEDLE

Pečivo šunka, zelenina

7 min

Postup prípravy:

Pečivo obložte uvedenými ingredienciami a konzumujte spolu so zeleninou. Dobrú chuť.

Ďalšie ingrediencie:
štipka zeleninovej soli (ak na prísady nie ste alergická)

ENERGIA	SACHARIDY	TUKY	BIELKOVINY
391.65 kcal	42.4 g	12.78 g	24.13 g
Ideál: 391.5 kcal	Ideál: 42.97 g	Ideál: 12.63 g	Ideál: 23.87 g

INGREDIENCIE

1 a ½ x krajec chlieb celozrnný ražný (ideálne kváskový) (75 g)
2 x plátok hydinová šunka (40 g)
2 a ½ ČL horčica plnotučná napr. Snico (22.5 g)
1 ks paprika žltá (75 g)
1 a ½ x plátok (9×9 cm) syr eidam, 45% t. v s. neúdený (33 g)

ROZLOŽENIE ENERGIE

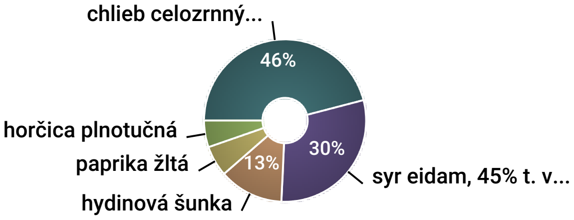

ĎALŠIE INFORMÁCIE O JEDLE

21 Vysoká GL   59 Chol

Avokádové smoothie

8 min

Postup prípravy:

Avokádo ošúpte a rozštvrťte. Potom spolu s ostatnými ingredienciami vložte do mixéra. Ak je smoothie husté, pridajte trochu vody. Dobrú chuť.

Ďalšie ingrediencie:

niekoľko lístkov 5-10 mäta, limetka zázvor podľa chuti (ak na prísady nie ste alergická)

ENERGIA	SACHARIDY	TUKY	BIELKOVINY
384.52 kcal	41.98 g	13.31 g	21.62 g
Ideál: 391.5 kcal	Ideál: 42.97 g	Ideál: 12.63 g	Ideál: 23.87 g

INGREDIENCIE

- *2 a ½ x čajová lyžička avokádo (37.5 g)*
- *½ ks banány (65 g)*
- *65 g kompót čučoriedkový*
- *2 a ½ dl mlieko polotučné (250 g)*
- *½ x dávka vegánsky proteín napr. Weider brownie chocolate (15 g)*

ROZLOŽENIE ENERGIE

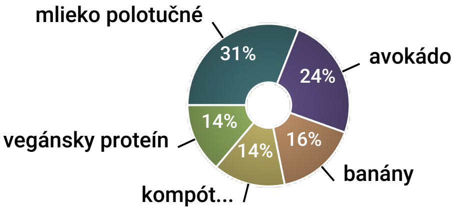

ĎALŠIE INFORMÁCIE O JEDLE

Quinoa kaša

15 min

Postup prípravy:

Do kastrólika nasypte quinoa vločky a pridajte vodu (podľa potreby, nemusíte sa riadiť množstvom vody, ktoré káže recept). Povarte. Stačí chvíľu, kaša bude rýchlo hustá. Ak chcete hustejšiu, povarte dlhšie, ak redšiu, pridajte vodu. Kašu dochuťte uvedenými ingredienciami. V prípade, že sa vo variante nachádza mlieko, vypite ho (nepoužije sa na prípravu receptu). Ak nemáte quinoa vločky, použite celú quinou, bude to trvať len o chvíľu dlhšie. Dobrú chuť.

Ďalšie ingrediencie:

tekvicové korenie, muškátový orech, mletý sušený zázvor (štipka), mleté klinčeky, mleté korenie, sypká stévia, soľ - z každého z korení len medzi prsty (varte spolu s quinoou) (ak na prísady nie ste alergická)

ENERGIA	SACHARIDY	TUKY	BIELKOVINY
392.77 kcal	47.02 g	11.64 g	22.39 g
Ideál: 391.5 kcal	Ideál: 42.97 g	Ideál: 12.63 g	Ideál: 23.87 g

INGREDIENCIE

- *2 a ½ PL quinoa (62.5 g)*
- *120 ml voda (120 g)*
- *½ x dávka vegánsky proteín napr. Weider brownie chocolate (15 g)*
- *1 x čajová lyžička živočíšne maslo (7 g)*

NÁPOJE

- *1 dl sójové mlieko (100 g)*

ROZLOŽENIE ENERGIE

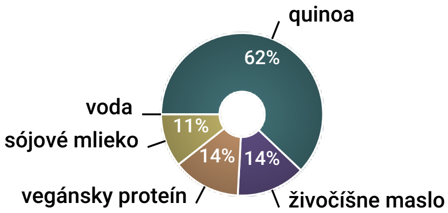

ĎALŠIE INFORMÁCIE O JEDLE

Vytvorené pomocou aplikácie planeat

Rybacie raňajky

7 min

Postup prípravy:

Z masla a rýb si v kľude spravte pomazánku a rovnomerne naneste na pečivo. Konzumujte spolu s vybranou zeleninou. Dobrú chuť.

Ďalšie ingrediencie:
bylinky, korenie, štipka zeleninovej soli, medvedí cesnak, vňať (ak na prísady nie ste alergická)

ENERGIA	SACHARIDY	TUKY	BIELKOVINY
401.82 kcal	47 g	12.62 g	22.38 g
Ideál: 391.5 kcal	Ideál: 42.97 g	Ideál: 12.63 g	Ideál: 23.87 g

INGREDIENCIE

1 x malá konzerva tuniak vo vlastnej šťave napr. Rio Mare (56 g)
2 x krajec chlieb celozrnný pšeničný (ideálne kváskový) (100 g)
2 a ½ x ČL živočíšne maslo (12.5 g)

ROZLOŽENIE ENERGIE

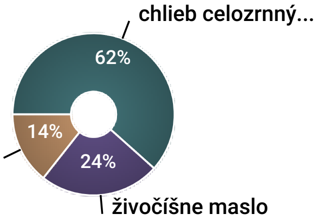

ĎALŠIE INFORMÁCIE O JEDLE

47 Chol

Banánovo-jahodový nápoj

5 min

Postup prípravy:

Ovocie spolu s mliekom a ostatnými prísadami rozmixujte a nápoj vypite.

Ďalšie ingrediencie:
stévia (ak na prísady nie ste alergická)

ENERGIA	SACHARIDY	TUKY	BIELKOVINY
399.39 kcal	47.62 g	12.64 g	21.13 g
Ideál: 391.5 kcal	Ideál: 42.97 g	Ideál: 12.63 g	Ideál: 23.87 g

INGREDIENCIE

1 menší kus banány (65 g)
3 dl sójové mlieko (300 g)
1 PL džem jahodový (37 g)
1 ČL olivový olej (5 g)
4 PL tvaroh jemný hrudkový 2,5% napr. Pilos (68 g)

ROZLOŽENIE ENERGIE

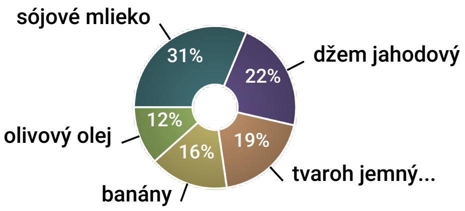

ĎALŠIE INFORMÁCIE O JEDLE

27 Vysoká GL

9.5 Chol

Vajce na tvrdo s pečivom a zeleninou

7 min

Postup prípravy:

Vajce na tvrdo konzumujte spolu s ostatnými ingredienciami a zeleninovou oblohou. Dobrú chuť.

Ďalšie ingrediencie:

štipka zeleninovej soli (ak na prísady nie ste alergická)

ENERGIA	SACHARIDY	TUKY	BIELKOVINY
390.66 kcal	42.91 g	12.61 g	23.76 g
Ideál: 391.5 kcal	Ideál: 42.97 g	Ideál: 12.63 g	Ideál: 23.87 g

INGREDIENCIE

- *1 a ½ x ks vajce na tvrdo (82.5 g)*
- *1 a ½ x kus rožky grahamové (63 g)*
- *1 ks paprika žltá (75 g)*
- *2 x plátok bravčová šunka (34 g)*

ĎALŠIE INFORMÁCIE O JEDLE

374 Chol

ROZLOŽENIE ENERGIE

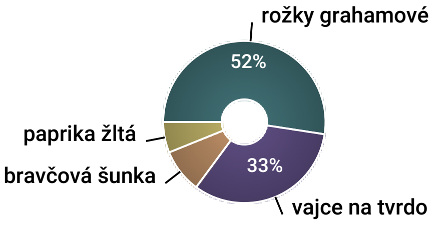

Cottage syr s pestom, zeleninou a pečivom

5 min

Postup prípravy:

Do cottage syru vmiešajte pesto. Premiešaný syr s pestom rovnomerne naneste na pečivo. Konzumujte spolu s nakrájanou zeleninou. Dobrú chuť.

Ďalšie ingrediencie:

bylinky (kurkuma, medvedí cesnak, bazalka), koreniny, pažítka (ak na prísady nie ste alergická)

ENERGIA	SACHARIDY	TUKY	BIELKOVINY
392.9 kcal	37.17 g	14.38 g	26.04 g
Ideál: 391.5 kcal	Ideál: 42.97 g	Ideál: 12.63 g	Ideál: 23.87 g

INGREDIENCIE

- *1 x balenie cottage cheese biely napr. Rajo (180 g)*
- *1 x ČL pesto bazalkové BIO (9 g)*
- *1 x krajec chlieb rascový (50 g)*
- *½ ks rajčiny (37.5 g)*
- *3 ks reďkovka (51 g)*

ĎALŠIE INFORMÁCIE O JEDLE

27 Chol

ROZLOŽENIE ENERGIE

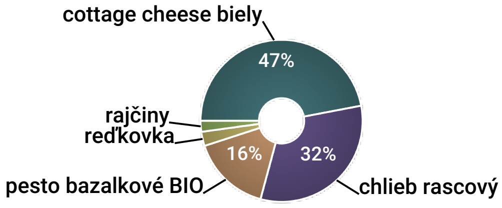

Banánovo-pohánkové lievance

15 min

Postup prípravy:

Večer pohánku zalejte vodou a dajte na noc do chladničky. Ráno prelejte cez sitko a dajte do mixéra. Pridajte bielka (alebo celé vajce, podľa toho ako uvádza recept), banán a mlieko a rozmixujte na homogénnu zmes. Rozohrejte panvicu, omastite zľahka olejom a pridajte dávku cesta. Pečte lievance. Podávajte ozdobené džemom alebo mliečnym dezertom uvedeným v recepte. Dobrú chuť.

Ďalšie ingrediencie:

škorica, soľ, stévia niekoľko kvapiek podľa chuti (ak na prísady nie ste alergická)

ENERGIA	SACHARIDY	TUKY	BIELKOVINY
375.57 kcal	44.26 g	12.49 g	19.02 g
Ideál: 391.5 kcal	Ideál: 42.97 g	Ideál: 12.63 g	Ideál: 23.87 g

INGREDIENCIE

3 x ks vaječný bielok (99 g)
1 menší kus banány (65 g)
1 x PL pohanka nelúpaná (neuvarená) (25 g)
½ dl mlieko polotučné 1,5% (50 g)
½ x balenie tvarohový dezert Termix (45 g)
2 x ČL kokosový olej (10 g)

ROZLOŽENIE ENERGIE

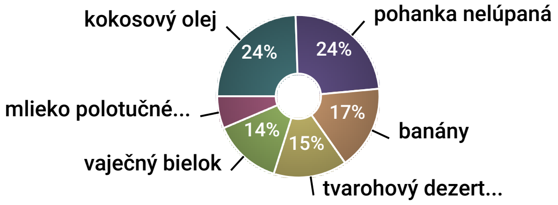

ĎALŠIE INFORMÁCIE O JEDLE

Obed

Tekvicové lečo

30 min

Postup prípravy:

Na panvici zohrejte olej. Všetku zeleninu očistite, nakrájajte na požadované kúsky a dajte restovať. Najprv cibuľku, cesnak a následne aj ostatnú zeleninu. Keď trochu zmäkne podlejte ju ešte pohárom vody. Ochuťte soľou, čiernym mletým korením, majoránkou alebo oreganom, prípadne podľa chuti môžete pridať aj chilli. Podávajte s vajíčkom uvareným na tvrdo (a/alebo cottage syrom) a pokiaľ recept uvádza aj s pečivom alebo ryžou.

Ďalšie ingrediencie:

štipka soli, čierne mleté korenie, oregano, majoránka, chilli (ak na prísady nie ste alergická)

ENERGIA	SACHARIDY	TUKY	BIELKOVINY
426.06 kcal	48.83 g	15.65 g	19.59 g
Ideál: 417.48 kcal	Ideál: 45.82 g	Ideál: 15.71 g	Ideál: 20.36 g

INGREDIENCIE

- *1 strúčik cesnak (3 g)*
- *½ x priemerný kus tekvica (125 g)*
- *½ ks paprika červená (37.5 g)*
- *½ ks paprika žltá (37.5 g)*
- *½ x kus (40 cm) jarná cibuľka (22 g)*
- *1 ČL repkový olej rafinovaný (5 g)*
- *1 a ½ x krajec chlieb celozrnný pšeničný (ideálne kváskový) (75 g)*
- *1 a ½ x ks vajce na tvrdo (82.5 g)*

ROZLOŽENIE ENERGIE

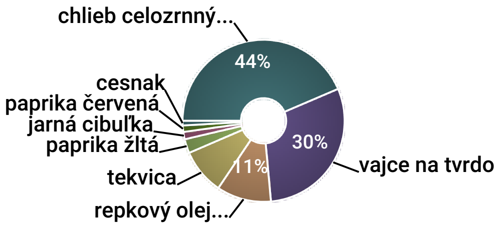

ĎALŠIE INFORMÁCIE O JEDLE

350 Chol

Polievka

Postup prípravy:

Polievku konzumujte ako hlavné jedlo.

ENERGIA	SACHARIDY	TUKY	BIELKOVINY
369.29 kcal	42.73 g	13.5 g	16.72 g
Ideál: 426 kcal	Ideál: 46.76 g	Ideál: 16.03 g	Ideál: 20.78 g

INGREDIENCIE

- *1 a ½ x porcia polievka z červenej šošovice (495 g) (☰ postup prípravy nižšie)*

ROZLOŽENIE ENERGIE

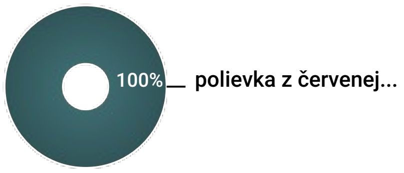

ĎALŠIE INFORMÁCIE O JEDLE

Stredná 10 GL

0 Chol

POSTUP PRÍPRAVY - POLIEVKA Z ČERVENEJ ŠOŠOVICE (3 x porcia = 990g)

120 g šošovica červená napr.Country life
1 a ½ PL olivový olej (21.6 g)
1 ks mrkva (153 g)
2 x porcia ľahký zeleninový vývar (693 g)

Postup prípravy: Šosovicu preperte v hrnci v studenej vode a zlejte ju. Mrkvu nastrúhajte najemno a osmažte na olivovom oleji. Potom pridajte červenú šošovicu, soľ, vývar a poduste. Pridajte rascu a kurkumu. Po uvarení tyčovým mixérom rozmixujte, pridajte chilli a podávajte so štipkou zeleného petržlenu.

pomocné ingrediencie: štipka soli, kurkuma, razca, chilli, šťava z limetky (v prípade, že na prísady nie ste alergický/á)

Plané mäso s prílohou a so zeleninou

🕒 20 min

Postup prípravy:

Okorenené kuracie/morčacie prsia opečte na oleji (masti, masle). Podľa druhu zeleniny ju buď orestujte na panvici, alebo zohrejte v rúre (max. 180 °C). Ľubovoľne dochuťte. Konzumujte spolu s prílohou. Dobrú chuť.

Ďalšie ingrediencie:

zeleninová soľ, korenie, bylinky (ak na prísady nie ste alergická)

ENERGIA	SACHARIDY	TUKY	BIELKOVINY
418.72 kcal	45.37 g	16.01 g	20.43 g
Ideál: 417.48 kcal	Ideál: 45.82 g	Ideál: 15.71 g	Ideál: 20.36 g

INGREDIENCIE

70 g morčacie prsia bez kosti
1 a ½ väčšia naberačka ryža varená napr. basmati, Vitana (90 g)
½ ks rajčiny (37.5 g)
2 a ½ ČL repkový olej rafinovaný (12.5 g)
1 x porcia zemiaková polievka (330 g) (☰ postup prípravy nižšie)

ROZLOŽENIE ENERGIE

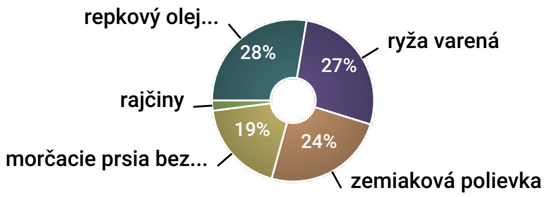

ĎALŠIE INFORMÁCIE O JEDLE

42 Chol

POSTUP PRÍPRAVY - ZEMIAKOVÁ POLIEVKA (6 x porcia = 2297g)

6 x stredný kus zemiaky neskoré (540 g)
1 x stredne veľká cibuľa (75 g)
2 ks mrkva (170 g)
1 PL olivový olej (12 g)
1500 ml voda pitná (1500 g)

Postup prípravy: Cibuľu, mrkvu, zemiaky očistite a nakrájajte. V hrnci rozohrejte olej a vložte cibuľu a mrkvu. Chvíľu orestujte a pridajte pokrájané zemiaky. Zalejte vodou, osoľte a okoreňte. Varte do zmäknutia zemiakov.

Pomocné ingrediencie: štipka soli, (štipka čierneho korenia) jedine ak máte odskúšané. Čierne korenie vo väčšom množstve môže spôsobovať problémy

Zemiakovo - kelové komíny s cottage syrom

150 min

Postup prípravy:

Zemiakovo - kelové komíny pripravte podľa návodu nižšie. Konzumujte spolu s cottage syrom a troškou oleja/masla pokiaľ máte predpísané. Dobrú chuť.

Ďalšie ingrediencie:

soľ (ak na prísady nie ste alergická)

ENERGIA	SACHARIDY	TUKY	BIELKOVINY
425.7 kcal	46.73 g	16.08 g	20.61 g
Ideál: 421.74 kcal	Ideál: 46.29 g	Ideál: 15.87 g	Ideál: 20.57 g

INGREDIENCIE

- *½ x balenie cottage cheese biely napr. Pilos (100 g)*
- *1 x ČL živočíšne maslo (5 g)*
- *1 a ½ x kus zemiakovo-kelové komíny (127.5 g) (☰ postup prípravy nižšie)*
- *1 x porcia polievka hrachovo-mrkvová (330 g) (☰ postup prípravy nižšie)*

ROZLOŽENIE ENERGIE

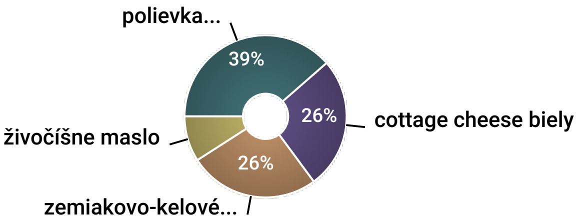

ĎALŠIE INFORMÁCIE O JEDLE

12 Stredná GL

42 Chol

POSTUP PRÍPRAVY - ZEMIAKOVO-KELOVÉ KOMÍNY (12 x kus = 1033g)

- *9 x stredný kus zemiaky neskoré (810 g)*
- *3 strúčik cesnak (9 g)*
- *1 x stredne veľká červená cibuľa (93.75 g)*
- *100 x g kel kučeravý*
- *20 g petržlen - vňať*

Postup prípravy: Zemiaky očistite a v šupke uvarte cca 40 minút vo vode do zmäknutia. Nechajte vychladnúť, aby ste ich mohli ošúpať. Medzitým si dobre očistite a nasekajte na drobno kel (podľa možnosti nie ružičkový), ktorý je dobré pred pečením spariť. Dajte do hrnca niekoľko lyžíc vody, nakrájaný kel a priveďte do varu. Poduste 3-5 minút, potom zlejte a opláchnite studenou vodou. Cibuľu nasekajte na drobno, cesnak očistite a nadrvte.
Rúru predhrejte na 175 ° C. Vychladnuté zemiaky rozmixujte ručným mixérom alebo roztlačte (nechajte kúsky, výsledok bude chutnejší), vmiešajte sparený kel, nasekanú cibuľu, štipku soli, cesnak a nasekané petržlenové lístky. Na plech dajte papier na pečenie a ideálne cez kruhovú formičku plňte do stĺpikov (ideálne 12), ľahko vždy stlačte. Pečte 40 minút.

POSTUP PRÍPRAVY - POLIEVKA HRACHOVO-MRKVOVÁ (7 x porcia = 2600g)

- *200 g hrach sušený*
- *2 ks mrkva (200 g)*
- *100 g cibuľa*
- *50 g cesnak*
- *50 g živočíšne maslo*
- *2000 ml voda pitná (2000 g)*

Postup prípravy: Deň vopred namočený hrach prepláchnite a vložte do hrnca s vodou variť. Počas varenia hrach nemiešajte, len občas potraste hrncom, aby sa hrach rovnomerne uvaril. Keď je hrach polomäkký, pridajte nakrájanú mrkvu, alebo inú tolerovanú zeleninu s cibuľou a uvarte do mäkka. Ponorným mixérom rozmixujte, až potom osoľte a dochuťte čerstvým cesnakom (poprípade majoránkou). Zjemnite maslom

Vytvorené pomocou aplikácie planeat

Losos so zeleninovým pyré

🕒 40 min

Postup prípravy:

Zemiaky a/alebo cviklu uvarte do mäkka (alebo kúpte už varenú, vákuovo balenú). Dajte do mixéra spolu s avokádom, štipkou soli, čiernym mletým korením a rozmixujte na pyré. Ak by bolo pyré husté, pridajte trochu mlieka. Lososa jemne posoľte a preložte do alobalu. Na plátok lososa poukladajte nakrájaný citrón a zabaľte. Ak recept uvádza aj cherry paradajky, pridajte ich k lososu nakrájané na polovice. Pečte vo vyhriatej rúre pri 180 stupňoch približne 20 minút. Dobrú chuť.

Ďalšie ingrediencie:
soľ, čierne mleté korenie (ak na prísady nie ste alergická)

ENERGIA	SACHARIDY	TUKY	BIELKOVINY
420.9 kcal	45.03 g	16.34 g	20.56 g
Ideál: 421.74 kcal	Ideál: 46.29 g	Ideál: 15.87 g	Ideál: 20.57 g

INGREDIENCIE

1 x malý kus zemiaky skoré (60 g)
1 stredný kus batáty sladké zemiaky (90 g)
2 x čajová lyžička avokádo (30 g)
80 g losos obyčajný
1 kus citróny (120 g)
5 ks cherry paradajky (60 g)

ROZLOŽENIE ENERGIE

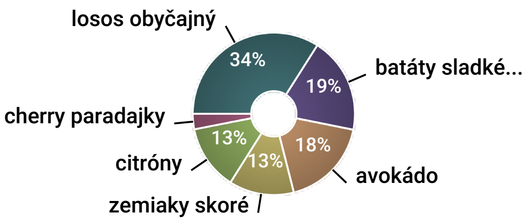

ĎALŠIE INFORMÁCIE O JEDLE

18 Stredná GL   42 Chol

Rizoto so sušenými paradajkami

🕒 45 min

Postup prípravy:

Nadrobno nakrájanú cibuľu orestujte na tuku do sklovita. Pridajte neumytú ryžu, ktorú orestujte asi 1 minútu. Postupne podlievajte vývarom, aby bola ryža zakrytá. Varte asi 18-20 minút, nie úplne do mäkka. Ak by bolo vývaru málo, podlievajte už len vodou. Rizoto dochuťte nastrúhaným parmezánom (len ak ho recept uvádza). Rizoto by malo byť krémové. Naservírujte do hlbokého taniera, ozdobte sušenými paradajkami a cottage syrom (len ak ho recept uvádza). Dobrú chuť.

Ďalšie ingrediencie:
soľ, biele víno, bazalka (ak na prísady nie ste alergická)

ENERGIA	SACHARIDY	TUKY	BIELKOVINY
427.21 kcal	46.31 g	16.71 g	20 g
Ideál: 426 kcal	Ideál: 46.76 g	Ideál: 16.03 g	Ideál: 20.78 g

INGREDIENCIE

2 ČL sušené paradajky (mleté) napr. Carthage garden (20 g)
½ x stredne veľká cibuľa (37.5 g)
45 g ryža jasmínová natural napr. Biolienka (neuvarená)
1 ČL repkový olej rafinovaný (5 g)
65 x g cottage cheese biely napr. Tesco
½ x šálka(250ml) polievka slepačia (bez rezancov) (125 g) (☰ postup prípravy nižšie)

ROZLOŽENIE ENERGIE

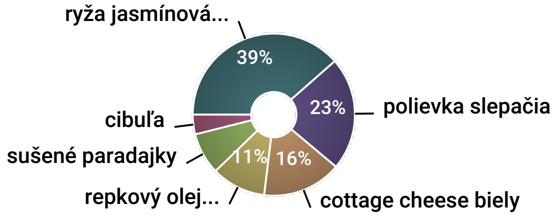

ĎALŠIE INFORMÁCIE O JEDLE

POSTUP PRÍPRAVY - POLIEVKA SLEPAČIA (BEZ REZANCOV) (19 x šálka(250ml) = 4858g)

½ x kus sliepka domáca (1500 g)
3000 ml voda (3000 g)
1 x stredne veľký petržlen (60 g)
1 x priemerný kus zeler bulvový (50 g)
1 x stredne veľká cibuľa (75 g)
1 strúčik cesnak (3 g)
2 ks mrkva (170 g)

Postup prípravy: 4 ks nové korenie
4 ks mleté čierne korenie
petržlenová vňať
soľ

Slepačie mäso očistite a spolu s kosťami vložte do studenej vody. Pridajte očistenú zeleninu a koreniny a varte do mäkka, cca 2 hodiny. Po uvarení mäkka a zeleniny, polievku precedte. Slepačie mäso a mrkvu pokrájajte na drobné kúsky a spolu s pokrájanou petržlenovou vňaťou alebo pažítkou vložte do polievky. V prípade potreby dochuťte.

Basmati rizoto s pečenou špargľou a lososom

30 min

Postup prípravy:

Filety lososa umyte, osoľte a dajte do parného hrnca na 15 min alebo do vody a povarte. Nakrájajte špargľu a opražte na masle do chrumkava. Ryžu chvíľku popražte, zalejte mliekom zmiešaným s vodou a povarte domäkka. Potom pridajte parmezán a miešajte asi 3 min. Pridajte pečenú špargľu, kúsky lososa a trochu masla na chuť. Dobú chuť.

Ďalšie ingrediencie:
štipka soli (ak na prísady nie ste alergická)

ENERGIA	SACHARIDY	TUKY	BIELKOVINY
424.31 kcal	46.13 g	16.48 g	19.97 g
Ideál: 421.74 kcal	Ideál: 46.29 g	Ideál: 15.87 g	Ideál: 20.57 g

INGREDIENCIE

50 x g ryža (neuvarená) Basmati
3 x kus (25 cm dĺžka) špargľa (63 g)
55 g losos obyčajný
1 dl mlieko polotučné (100 g)
1 a ½ x čajová lyžička živočíšne maslo (10.5 g)

ROZLOŽENIE ENERGIE

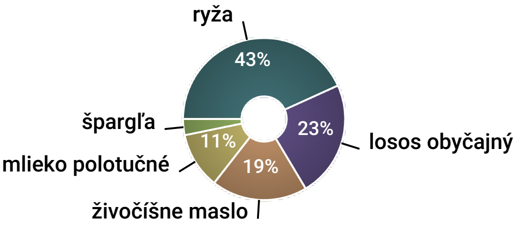

ĎALŠIE INFORMÁCIE O JEDLE

Červená šošovica s pečenou cviklou a syrom

30 min

Postup prípravy:

Šošovicu uvarte domäkka. Nakrájajte na kolieska červenú repu (cviklu) a upečte v rúre. Zmiešajte so šošovicou, pridajte strúhaný petržlen, cesnak, pór a olej. Pridajte syr.

Ďalšie ingrediencie:

kôpor, majoránka, štipka soli (ak na prísady nie ste alergická)

ENERGIA	SACHARIDY	TUKY	BIELKOVINY
423.87 kcal	44.43 g	14.81 g	25.36 g
Ideál: 421.74 kcal	Ideál: 46.29 g	Ideál: 15.87 g	Ideál: 20.57 g

INGREDIENCIE

½ strúčik cesnak (1.5 g)
1 x stredne veľký petržlen (60 g)
½ x (10cm dĺžka, 10 cm obvod) pór (34.5 g)
45 g šošovica červená napr.Country life
2 x malý kus červená repa (cvikla) (100 g)
2 ČL olivový olej (10 g)
½ x balenie cottage cheese biely napr. Rajo (90 g)

ROZLOŽENIE ENERGIE

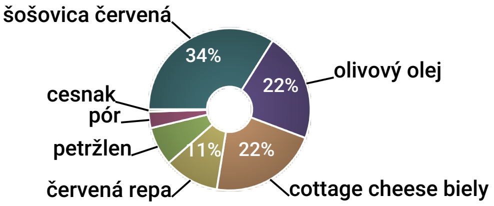

ĎALŠIE INFORMÁCIE O JEDLE

14 Chol

Pstruh s prílohou

45 min

Postup prípravy:

Pripravte si suroviny. Pstruha umyte a osušte. Z každej strany ho osoľte a okoreňte čiernym korením. Vložte ho do zapekacej sklenenej misky (jenské sklo). Pstruha zo spodnej časti narežte a dovnútra vložte niekoľko plátkov citrónu, petržlenovej vňate, masla, a tymiánu. Niekoľko plátkov citrónu si nechajte aj na vrch a pridajte nakrájané plátky cesnaku. Pečte vo vyhriatej trúbe asi na 180°C približne 30 min. Konzumujte spolu s prílohou. Dobrú chuť.

Ďalšie ingrediencie:

petržlenová vňať, korenie, tymián (ak na prísady nie ste alergická)

ENERGIA	SACHARIDY	TUKY	BIELKOVINY
424.81 kcal	48.28 g	15.12 g	21.04 g
Ideál: 417.48 kcal	Ideál: 45.82 g	Ideál: 15.71 g	Ideál: 20.36 g

INGREDIENCIE

90 g pstruh dúhový
2 x väčšia naberačka batáty sladké zemiaky (120 g)
½ x kus citróny (60 g)
1 x ČL živočíšne maslo (5 g)
1 x porcia zemiaková polievka (330 g) (☰ postup prípravy nižšie)

ROZLOŽENIE ENERGIE

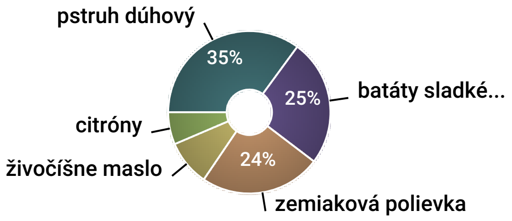

ĎALŠIE INFORMÁCIE O JEDLE

20 Vysoká GL   75 Chol

POSTUP PRÍPRAVY - ZEMIAKOVÁ POLIEVKA (6 x porcia = 2297g)

6 x stredný kus zemiaky neskoré (540 g)
1 x stredne veľká cibuľa (75 g)
2 ks mrkva (170 g)
1 PL olivový olej (12 g)
1500 ml voda pitná (1500 g)

Postup prípravy: Cibuľu, mrkvu, zemiaky očistite a nakrájajte. V hrnci rozohrejte olej a vložte cibuľu a mrkvu. Chvíľu orestujte a pridajte pokrájané zemiaky. Zalejte vodou, osoľte a okoreňte. Varte do zmäknutia zemiakov.

Pomocné ingrediencie: štipka soli, (štipka čierneho korenia) jedine ak máte odskúšané. Čierne korenie vo väčšom množstve môže spôsobovať problémy

Prívarok zo zelenej fazuľky s vajíčkom

30 min

Postup prípravy:

Zelenú fazuľku dajte do hrnca, zalejte vodou a asi 15 minút povarte. Potom vodu zlejte. Umyté zemiaky nakrájajte na kocky a tiež uvarte do mäkka. Po uvarení vodu sceďte. Zemiaky preložte opäť do hrnca s vriacou vodou tak aby boli všetky podliate. Zemiaky mixujte tyčovým mixérom a prilievajte vodu podľa toho, aký hustý chcete prívarok mať (ak sa Vám zdá zemiakov veľa, niektoré môžete nechať vcelku a primiešať do prívarku nakoniec spolu s fazuľkou). Keď ste s hustotou spokojný prilejte mlieko a prívarok ešte chvíľu povarte už aj spolu s uvarenými fazuľkami. Podľa chuti pridajte soľ, čierne mleté korenie a ocot (prípadne mletú červenú paprika, kôpor alebo štipku cukru). Podávajte s vajíčkom natvrdo a chlebom.

Ďalšie ingrediencie:

soľ, čierne mleté korenie, mletá červená paprika, kôpor, cukor, ocot (ak na prísady nie ste alergická)

ENERGIA	SACHARIDY	TUKY	BIELKOVINY
404.21 kcal	44.16 g	14.69 g	21.12 g
Ideál: 404.7 kcal	Ideál: 44.42 g	Ideál: 15.23 g	Ideál: 19.74 g

INGREDIENCIE

- *70 x g fazuľka*
- *1 dl mlieko polotučné 1,5% (100 g)*
- *2 x ks vajce na tvrdo (110 g)*
- *1 x malý kus zemiaky neskoré (60 g)*
- *1 x krajec chlieb pohánkový (50 g)*

ROZLOŽENIE ENERGIE

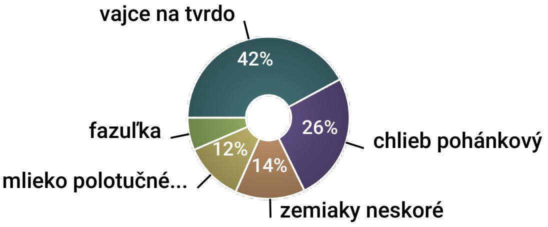

ĎALŠIE INFORMÁCIE O JEDLE

471 Chol

Cestoviny s tofu a zeleninou

🕒 25 min

Postup prípravy:

Tofu nakrájajte na kocky, pokropte sójovou omáčkou (ak na ňu nie ste alergický/á) a nechajte asi 10 min odstáť. Medzitým si na na oleji speňte nakrájanú cibuľu, rozotretý cesnak (iba ak sú v recepte uvedené). Mrkvu a cukinu umyte, očistite a nastrúhajte na hrubšie pásiky. Zeleninu spolu s nakrájaným tofu pridajte na panvicu, premiešajte a duste asi 7 min za občasného miešania. Pridajte uvarené cestoviny. Dobrú chuť.

Ďalšie ingrediencie:
sójová omáčka, soľ, mleté čierne korenie, (ak na prísady nie ste alergická)

ENERGIA	SACHARIDY	TUKY	BIELKOVINY
425.4 kcal	47.18 g	15.84 g	20.63 g
Ideál: 426 kcal	Ideál: 46.76 g	Ideál: 16.03 g	Ideál: 20.78 g

INGREDIENCIE

- *40 g tofu biele napr. Alfa Bio*
- *2 ČL olivový olej (10 g)*
- *1 x väčšia naberačka cestoviny dvojvaječné varené (60 g)*
- *½ x kus (18cm dĺžka, 13,5 cm obvod v strede) cukina (93.5 g)*
- *½ ks mrkva (42.5 g)*
- *1 x porcia hrachová polievka (330 g) (☰ postup prípravy nižšie)*

ROZLOŽENIE ENERGIE

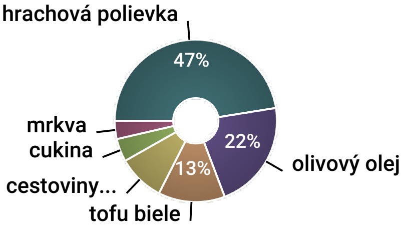

ĎALŠIE INFORMÁCIE O JEDLE

16 Stredná GL

POSTUP PRÍPRAVY - HRACHOVÁ POLIEVKA (5 x porcia = 1945g)

- *2 x stredný kus zemiaky neskoré (180 g)*
- *250 x g hrach sušený*
- *1 PL olivový olej (12 g)*
- *1 strúčik cesnak (3 g)*
- *1500 ml voda pitná (1500 g)*

Postup prípravy: Do hrnca si dajte rozohriať olej, na ktorom opečte nakrájaný cesnak. Do hrnca si nasypte sušený hrach, očistené a nakrájané na malé kocky zemiaky, osoľte a zalejte vodou. Polievku varte, kým zemiaky a hrach nezmäknú a následne všetko rozmixujte do hladka.

Vytvorené pomocou aplikácie planeat

Rizoto s kuracím mäsom a zeleninou

🕒 60 min

Postup prípravy:

Ryžu uvarte v osolenej vode domäkka (približne 15 minút, podľa typu ryže). Uvarenú ryžu prepláchnite vlažnou vodou a nechajte odkvapkať. Očistenú mrkvu a stonkový zeler nakrájajte na malé kocky. Cesnak a chilli papričku zbavenú semienok nakrájajte nadrobno. Zeleninu opečte na rozohriatom tuku. Dochuťte štipkou soli, čiernym mletým korením. Keď zelenina trochu zmäkne, pridajte nakrájané šampiňóny a premiešajte. Chvíľu opekajte a pridajte aj nakrájanú cibuľku a na polovice nakrájané paradajky. Nakoniec pridajte uvarenú ryžu a umytý špenát. Zľahka premiešajte kým sa špenát sparí. Podávajte s pečeným kurčaťom. Dobrú chuť.

Ďalšie ingrediencie:
soľ, čierne mleté korenie, chilli paprička (ak na prísady nie ste alergická)

ENERGIA	SACHARIDY	TUKY	BIELKOVINY
413.09 kcal	45.19 g	15.34 g	20.76 g
Ideál: 413.22 kcal	Ideál: 45.35 g	Ideál: 15.55 g	Ideál: 20.16 g

INGREDIENCIE

½ x stonka zeler stonkový (16.5 g)
½ strúčik cesnak (1.5 g)
50 g ryža guľatozrnná natural napr. Biolienka (neuvarená)
½ ks mrkva (42.5 g)
2 ks šampiňóny čerstvé (52 g)
1 a ½ x čajová lyžička živočíšne maslo (10.5 g)
1 miska objem (300 ml) špenát (20 g)
2 ks cherry paradajky (24 g)
70 g Pečené kura (☰ postup prípravy nižšie)

ROZLOŽENIE ENERGIE

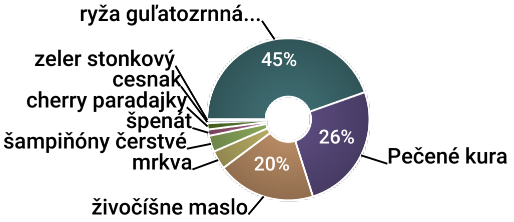

ĎALŠIE INFORMÁCIE O JEDLE

POSTUP PRÍPRAVY - PEČENÉ KURA (1012 g)

1000 g kurča domáce
1 PL olivový olej (12 g)

Postup prípravy: Umyté a osušené kurča dôkladne osoľte a okoreňte. Preložte do zapekacej misy, pokvapkajte olejom, posypte tymiánom a podlejte trochou vody. Pečte vo vyhriatej rúre pri 200 stupňoch približne 1 hodinu. Počas pečenia podlievajte kura vlastným výpekom. Upečené rozporciujte.

Cviklové rizoto

45 min

Postup prípravy:

Nadrobno nakrájaný zeler a cibuľu poduste na tuku. Ochuťte štipkou soli, čiernym mletým korením a bobkovým listom. Keď zelenina trochu zmäkne, primiešajte neumytú ryžu. Do rizota postupne po nabračkách pridávajte vývar, vždy aby bola ryža zaliata. Po chvíli primiešajte očistenú a nastrúhaná cviklu. Asi po 20 minútach (keď je ryža uvarená) pridajte trochu citrónovej šťavy a strúhaný parmezán (len ak ho recept uvádza). Zľahka premiešajte a nechajte odpočívať. Ak by ste mali málo vývaru a ryža by stále nebola uvarená, podlejte vodou. Na tanier naservírujte pripravené rizoto, pridajte kúsky mozarelly a sezamovými semenami (len ak ich máte v recepte uvedené). Dobrú chuť.

Ďalšie ingrediencie:
soľ, čierne mleté korenie, bobkový list, citrónová šťava, (ak na prísady nie ste alergická)

ENERGIA	SACHARIDY	TUKY	BIELKOVINY
419.59 kcal	47.08 g	15.46 g	20.2 g
Ideál: 413.22 kcal	Ideál: 45.35 g	Ideál: 15.55 g	Ideál: 20.16 g

INGREDIENCIE

- *½ x stonka zeler stonkový (16.5 g)*
- *½ x stredne veľká cibuľa (37.5 g)*
- *45 g ryža (neuvarená) Basmati*
- *1 malý kus červená repa (cvikla) (50 g)*
- *1 x čajová lyžička živočíšne maslo (7 g)*
- *4 plátok (20g) mozzarela light napr. GALBANI (80 g)*
- *½ x hrnček ľahký zeleninový vývar (125 g) (☰ postup prípravy nižšie)*

ROZLOŽENIE ENERGIE

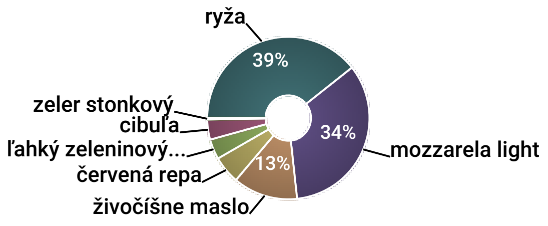

ĎALŠIE INFORMÁCIE O JEDLE

POSTUP PRÍPRAVY - ĽAHKÝ ZELENINOVÝ VÝVAR (10 x hrnček = 2609g)

- *1 PL olivový olej (12 g)*
- *3 x (10cm dĺžka, 10 cm obvod) pór (207 g)*
- *1 x stredne veľká cibuľa (75 g)*
- *3 ks mrkva (255 g)*
- *30 x g zeler - vňať*
- *2000 ml voda pitná (2000 g)*
- *30 g petržlen - vňať*

Postup prípravy: V polievkovom hrnci zohrejte olej. pridajte nadrobno pokrájaný pór a cibuľu, dobre premiešajte a oheň zmiernite. Prikryte natesno a 20 minút pomaly duste. Pridajte bobkový list, tymián, petržlenovú vňať, nadrobno pokrájanú mrkvu, zeler a soľ. Zalejte 2 litrami studenej vody a zosilnite oheň. Pomaly nechajte zovrieť a z povrchu počas varenia zbierajte penu a oheň znovu zmiernite. Prikryte a 35 minút pomaly varte. Vývar nalejte do veľkej ohňovzdornej nádoby a nechajte vychladnúť.

Vývar s rezancami

Postup prípravy:

Vývar konzumujte spolu s uvarenými rezancami. Dobrú chuť.

ENERGIA	SACHARIDY	TUKY	BIELKOVINY
411.06 kcal<br>Ideál: 426 kcal	46.34 g<br>Ideál: 46.76 g	14.41 g<br>Ideál: 16.03 g	21.24 g<br>Ideál: 20.78 g

INGREDIENCIE

60 g cestoviny dvojvaječné (neuvarené)
1 x šálka(250ml) polievka slepačia (bez rezancov) (250 g) (
☰ postup prípravy nižšie)

ĎALŠIE INFORMÁCIE O JEDLE

20 Vysoká GL

67 Chol

ROZLOŽENIE ENERGIE

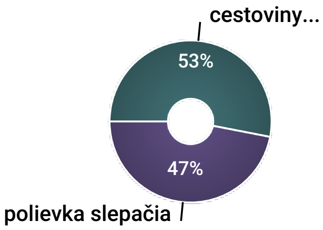

POSTUP PRÍPRAVY - POLIEVKA SLEPAČIA (BEZ REZANCOV) (19 x šálka(250ml) = 4858g)

½ x kus sliepka domáca (1500 g)
3000 ml voda (3000 g)
1 x stredne veľký petržlen (60 g)
1 x priemerný kus zeler bulvový (50 g)
1 x stredne veľká cibuľa (75 g)
1 strúčik cesnak (3 g)
2 ks mrkva (170 g)

Postup prípravy: 4 ks nové korenie
4 ks mleté čierne korenie
petržlenová vňať
soľ

Slepačie mäso očistite a spolu s kosťami vložte do studenej vody. Pridajte očistenú zeleninu a koreniny a varte do mäkka, cca 2 hodiny. Po uvarení mäkka a zeleniny, polievku precedte. Slepačie mäso a mrkvu pokrájajte na drobné kúsky a spolu s pokrájanou petržlenovou vňaťou alebo pažítkou vložte do polievky. V prípade potreby dochuťte.

Cestoviny so zeleninovou omáčkou

🕒 25 min

Postup prípravy:

Cestoviny uvarte podľa návodu na obale. Medzitým na tuku opražte nakrájanú slaninku (ak je uvedené v recepte), nadrobno nakrájanú cibuľku a cesnak. Následne pridajte paradajkové pyré a zeleninu. Nechajte povariť domäkka. Do omáčky pridajte tvaroh/lučinu, jemne osoľte a okoreňte a pridajte oregano. Omáčku podávajte spolu s uvarenými cestovinami, posypte parmezánom (ak ho máte v recepte uvedený). Dobrú chuť.

ENERGIA	SACHARIDY	TUKY	BIELKOVINY
434.54 kcal<br>Ideál: 426 kcal	51.05 g<br>Ideál: 46.76 g	15.23 g<br>Ideál: 16.03 g	20.38 g<br>Ideál: 20.78 g

INGREDIENCIE

2 a ½ ČL repkový olej rafinovaný (12.5 g)
1 strúčik cesnak (3 g)
½ x stredne veľká cibuľa (37.5 g)
2 a ½ x PL paradajkové pyré napr. Valfrutta (35 g)
70 g mrazený karfiol
3 a ½ PL tvaroh jemný hrudkový 2,5% napr. Pilos (59.5 g)
60 g cestoviny celozrnné, bezvaječné (neuvarené)

ROZLOŽENIE ENERGIE

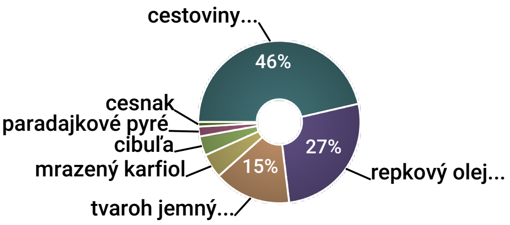

ĎALŠIE INFORMÁCIE O JEDLE

Francúzske zemiaky

🕒 60 min

Postup prípravy:

Zemiaky najskôr uvarte v šupke, potom očistite a nakrájajte na tenké plátky. Na dno pekáča natrite olej.Dajte prvú vrstvu zemiakov. Na ne trochu soli a čierneho korenia. Na to ukladajte vrstvu natvrdo uvarených vajec (bielkov), nakrájaných na tenké plátky. Pridajte plátky syra alebo strúhaný syr. Následne navrch naukladajte koliečka šunky. Zalejte smotanou. Vrstvy opakujte. Zakryte alobalom a dajte do trúby na 45-60 minút. Po ukončení ich popečte ešte asi 15 minút bez alobalu. Dobrú chuť.

Ďalšie ingrediencie:
štipka soli, čierne korenie (ak na prísady nie ste alergická)

ENERGIA	SACHARIDY	TUKY	BIELKOVINY
423.09 kcal	46.23 g	15.95 g	20.77 g
Ideál: 421.74 kcal	Ideál: 46.29 g	Ideál: 15.87 g	Ideál: 20.57 g

INGREDIENCIE

- *3 a ½ x väčšia naberačka batáty sladké zemiaky (210 g)*
- *1 a ½ ČL repkový olej rafinovaný (7.5 g)*
- *2 x veľkosť M slepačí bielok (74 g)*
- *½ x plátok (9×9 cm) syr eidam údený, 45% t. v s. (11 g)*
- *1 x plátok hydinová šunka (20 g)*
- *2 a ½ PL smotana kyslá pochúťková napr. Rajo (30 g)*
- *2 a ½ (8cm) ks uhorky zavárané kyslé napr. Bioline (85 g)*

ROZLOŽENIE ENERGIE

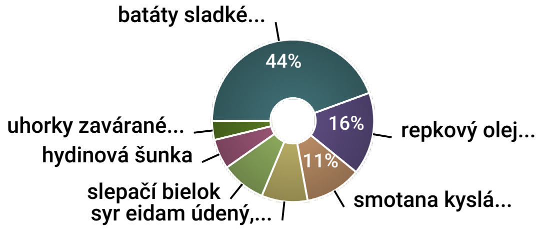

ĎALŠIE INFORMÁCIE O JEDLE

Hovädzie plátky s pórom a prílohou

🕒 60 min

Postup prípravy:

Mäso umyte, osušte, osoľte. Opečte na oleji (masle, masti) zo všetkých strán. Podlejte vodou a duste pod pokrievkou. Medzitým očistite a umyte pór. Po dĺžke ho rozrežte a nakrájajte na prúžky. Na panvici ho poduste, osoľte, okoreňte. Mäkké mäso nakrájajte na plátky, na tanieri obložte poduseným pórom. Konzumujte s prílohou.

Ďalšie ingrediencie:
soľ, korenie, bylinky (ak na prísady nie ste alergická)

ENERGIA	SACHARIDY	TUKY	BIELKOVINY
418.69 kcal	45.98 g	15.81 g	20.27 g
Ideál: 421.74 kcal	Ideál: 46.29 g	Ideál: 15.87 g	Ideál: 20.57 g

INGREDIENCIE

- *70 g hovädzie zadné - surové*
- *1 x ČL živočíšne maslo (5 g)*
- *½ x (10cm dĺžka, 10 cm obvod) pór (34.5 g)*
- *2 x väčšia naberačka ryža nelúpaná (varená) (120 g)*
- *1 x porcia brokolicová polievka (330 g) (☰ postup prípravy nižšie)*

ROZLOŽENIE ENERGIE

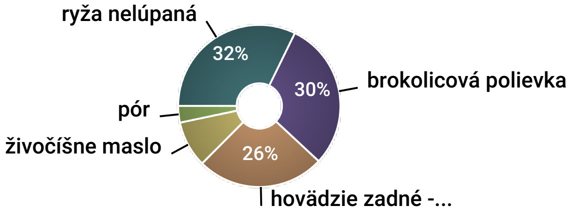

ĎALŠIE INFORMÁCIE O JEDLE

POSTUP PRÍPRAVY - BROKOLICOVÁ POLIEVKA (4 x porcia = 1377g)

1 x 1 balenie brokolica (500 g)
1 x stredný kus zemiaky neskoré (90 g)
1 x stredne veľká cibuľa (75 g)
2 dl mlieko polotučné 1,5% (200 g)
1 PL olivový olej (12 g)
500 ml voda pitná (500 g)

Postup prípravy: Cibuľu nakrájajte najemno a opražte ju na oleji v hrnci. Pridajte umytú, naružičkovanú brokolicu, na kocky nakrájaný zemiak a zalejte vodou tak, aby zelenina plávala. Osoľte, okoreňte a nechajte variť. Zhruba po 15 minútach, keď brokolica a zemiaky dostatočne zmäkli pridajte mlieko. Poriadne zamiešajte. Tyčovým mixérom rozmixujte celú zmes, podľa hustoty pridajte ešte mlieko. Opäť vráťte na oheň a priveďte k varu.

Pomocné ingrediencie: štipka soli, (štipka čierneho korenia) jedine ak máte odskúšané. Korenie najmä čierne vo väčšom množstve môže spôsobovať problémy

Sladko-pikantné karí z červenej šošovice s kuracím mäsom

45 min

Postup prípravy:

Cibuľu nakrájajte na malé kúsky a jemne opražte na oleji. Pridajte k nej nakrájané kuracie mäso a chvíľu opražte. Ďalej pridajte mrazenú zeleninu, okoreňte kari korením, zázvorom a čili (podľa chuti). Opražte 2 minúty. Pridaje opláchnutú šošovicu a zalejte vodou. Ak je zmes veľmi hustá, môžeme pridať viac vody. Varte 30 minút, alebo kým šošovica nezmäkne. Nakoniec pridajte kokosové mlieko a pasterizovaný med (len ak káže recept), kurkumu, osoľte a ešte chvíľku nechajte na miernom ohni variť. Podávajte s uvedenou prílohou.

Ďalšie ingrediencie:
soľ (ak na prísady nie ste alergická)

ENERGIA	SACHARIDY	TUKY	BIELKOVINY
433.88 kcal	50.48 g	14.74 g	21.9 g
Ideál: 421.74 kcal	Ideál: 46.29 g	Ideál: 15.87 g	Ideál: 20.57 g

INGREDIENCIE

½ x kus (40 cm) jarná cibuľka (22 g)
35 g mrazená zeleninová zmes jarná
35 g šošovica červená napr.Country life
2 a ½ ČL olivový olej (12.5 g)
2 g čili korenie
2 g korenie kari
2 g kurkuma
2 g ďumbier (zázvor)
30 g ryža nelúpaná (natural neuvarená)
30 g kuracie prsia bez kosti

ROZLOŽENIE ENERGIE

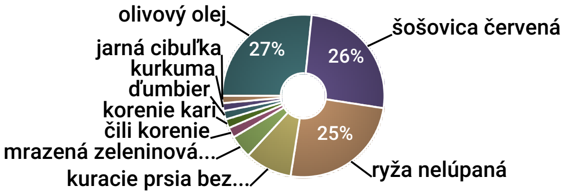

ĎALŠIE INFORMÁCIE O JEDLE

23 Chol

Krémová fazuľová polievka

🕒 100 min

Postup prípravy:

Fazuľu deň vopred namočte. Pred prípravou zlejte a zalejte čerstvou vodou a dajte variť. Pridajte nakrájaný petržlen a mrkvu. Všetko spolu nechajte variť na miernom ohni, až kým fazuľa dostatočne nezmäkne. Keď máte fazuľu uvarenú, naberačkou odoberte do taniera asi polovicu fazule a čo najviac mrkvy. Zvyšnú fazuľu a petržlen spolu s natrhanou mozzarelou (ak je uvedená v recepte) v hrnci rozmixujte ponorným mixérom na hladkú konzistenciu. Pridajte naspäť zvyšnú fazuľu a mrkvu, dochuťte čiernym korením, soľou, pridajte olej a pretlačený cesnak. Všetko zamiešajte a nechajte krátko prevariť. Pre viac porcií len roznásobte ingrediencie.

Ďalšie ingrediencie:
štipka soli, čierne korenie (ak na prísady nie ste alergická)

ENERGIA	SACHARIDY	TUKY	BIELKOVINY
421.12 kcal	48.85 g	13.77 g	22.63 g
Ideál: 421.74 kcal	Ideál: 46.29 g	Ideál: 15.87 g	Ideál: 20.57 g

INGREDIENCIE

70 x g fazuľa
½ strúčik cesnak (1.5 g)
½ ks mrkva (42.5 g)
½ x stredne veľký petržlen (30 g)
½ PL olivový olej (6 g)
30 g mozarela

ROZLOŽENIE ENERGIE

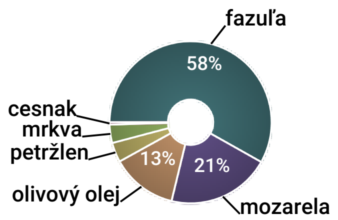

ĎALŠIE INFORMÁCIE O JEDLE

sportdiag

Menu - Obed

Rizoto z planého mäsa

Postup prípravy:

Rizoto ako menu variant v reštauračných zariadeniach. Tento recept si môžete spraviť aj doma, nie len ako menu variant.

Ďalšie ingrediencie:
korenie, zeleninová soľ, bylinky, huby, zelenina, tvrdý syr (posypať), zavárané uhorky (ak na prísady nie ste alergická)

ENERGIA	SACHARIDY	TUKY	BIELKOVINY
322.92 kcal<br>Ideál: 319.5 kcal	35.62 g<br>Ideál: 35.07 g	12.07 g<br>Ideál: 12.02 g	15.76 g<br>Ideál: 15.59 g

INGREDIENCIE

55 g morčacie prsia bez kosti
2 a ½ x väčšia naberačka ryža nelúpaná (varená) (150 g)
2 ČL repkový olej rafinovaný (10 g)

ĎALŠIE INFORMÁCIE O JEDLE

33 Chol

ROZLOŽENIE ENERGIE

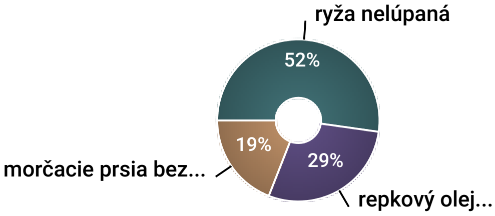

Bravčové mäso s prílohou

Postup prípravy:

Bravčové mäso s prílohou a zeleninovou oblohou ako menu variant v reštauračných zariadeniach. Ak máte na výber, vyberajte si predovšetkým listovú zeleninu s paradajkami, rukolou, špenátom, brokolicou. Zeleninovú zmes ako kukurica, hrach konzumujte menej, ale nevynechávajte z jedálnička. Ak Vám ponúknu poliať mäso mastnou omáčkou, zdvorilo odmietnite :) Túto kombináciu si môžete prichystať aj doma, nie len ako „menu variant“. Zeleninové prílohy konzumujte len také, na ktoré netrpíte alergiou. Dobrú chuť.

Ďalšie ingrediencie:
soľ, korenie, zeleninová obloha (ak na prísady nie ste alergická)

ENERGIA	SACHARIDY	TUKY	BIELKOVINY
358.08 kcal<br>Ideál: 362.1 kcal	38.82 g<br>Ideál: 39.74 g	13.32 g<br>Ideál: 13.63 g	18.3 g<br>Ideál: 17.66 g

INGREDIENCIE

70 g bravčová panenská sviečkovica
1 a ½ ČL repkový olej rafinovaný (7.5 g)
1 a ½ x väčšia naberačka zemiaky neskoré varené (90 g)
1 x porcia zemiaková polievka (330 g) (☰ postup prípravy nižšie)

ĎALŠIE INFORMÁCIE O JEDLE

46 Chol

ROZLOŽENIE ENERGIE

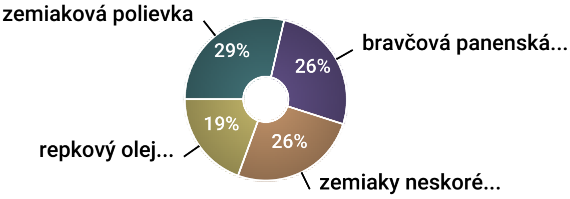

POSTUP PRÍPRAVY - ZEMIAKOVÁ POLIEVKA (6 x porcia = 2297g)

6 x stredný kus zemiaky neskoré (540 g)
1 x stredne veľká cibuľa (75 g)
2 ks mrkva (170 g)
1 PL olivový olej (12 g)
1500 ml voda pitná (1500 g)

Postup prípravy: Cibuľu, mrkvu, zemiaky očistite a nakrájajte. V hrnci rozohrejte olej a vložte cibuľu a mrkvu. Chvíľu orestujte a pridajte pokrájané zemiaky. Zalejte vodou, osoľte a okoreňte. Varte do zmäknutia zemiakov.

Pomocné ingrediencie: štipka soli, (štipka čierneho korenia) jedine ak máte odskúšané. Čierne korenie vo väčšom množstve môže spôsobovať problémy

Hovädzie, teľacie mäso s prílohou

Postup prípravy:

Hovädzie (resp. teľacie ak je uvedené vo variante) mäso s prílohou plus zelenina, ako menu variant v reštauračných zariadeniach. Ak Vám ponúknu poliať mäso mastnou omáčkou, zdvorilo odmietnite :) Túto kombináciu si môžete pripraviť aj doma, nie len ako „menu variant“. Dobrú chuť.

Ďalšie ingrediencie:
korenie, bylinky, soľ, zeleninová obloha (ak na prísady nie ste alergická)

ENERGIA	SACHARIDY	TUKY	BIELKOVINY
361.62 kcal	40.38 g	13.36 g	17.52 g
Ideál: 362.1 kcal	Ideál: 39.74 g	Ideál: 13.63 g	Ideál: 17.66 g

INGREDIENCIE

70 g teľacia sviečkovica
1 a ½ ČL repkový olej rafinovaný (7.5 g)
3 x väčšia naberačka zemiaky neskoré varené (180 g)

ĎALŠIE INFORMÁCIE O JEDLE

55 Chol

ROZLOŽENIE ENERGIE

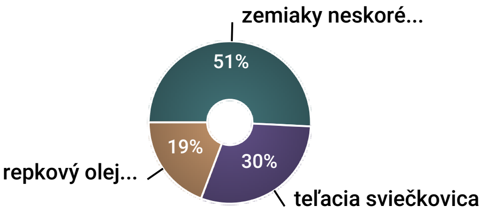

Olovrant

Polievka

Postup prípravy:

Polievku konzumujte ako hlavné jedlo.

ENERGIA	SACHARIDY	TUKY	BIELKOVINY
216.9 kcal<br>Ideál: 222 kcal	14.75 g	12.82 g	9.07 g

INGREDIENCIE

1 a ½ x porcia avokádovo-kokosová polievka z hovädzieho vývaru (495 g) (☰ postup prípravy nižšie)

ĎALŠIE INFORMÁCIE O JEDLE

Nízka 1.2 GL

0 Chol

ROZLOŽENIE ENERGIE

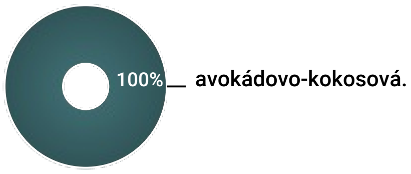

POSTUP PRÍPRAVY - AVOKÁDOVO-KOKOSOVÁ POLIEVKA Z HOVÄDZIEHO VÝVARU (4 x porcia = 1356g)

5 dl kokosové mlieko (500 g)
½ ks avokádo (97.5 g)
1 strúčik cesnak (3 g)
5 g ďumbier (zázvor)
3 x hrnček vývar z kostí (750 g)

Postup prípravy: Rozmixujeme všetko spolu do jemnej hmoty, zahrejeme / povaríme podľa potreby. Vhodná aj ako studená polievka.

pomocné ingrediencie: himalájska soľ (v prípade, že na prísady nie ste alergický/á)

Bagetka

⏱ 4 min

Postup prípravy:

Bagetku prekrojte na polovicu, naneste trošku masla, plátok šunky a ľubovoľnú zeleninu.

Ďalšie ingrediencie:

ľubovoľná zelenina (ak na prísady nie ste alergická)

ENERGIA	SACHARIDY	TUKY	BIELKOVINY
206.85 kcal<br>Ideál: 210.9 kcal	25.6 g	5.82 g	11.64 g

INGREDIENCIE

1 x kus rožky biele (42 g)
1 PL mäkký nezrejúci syr napr. Lučina (14 g)
2 x plátok priemer 14.5 cm morčacia šunka (36 g)

ĎALŠIE INFORMÁCIE O JEDLE

ROZLOŽENIE ENERGIE

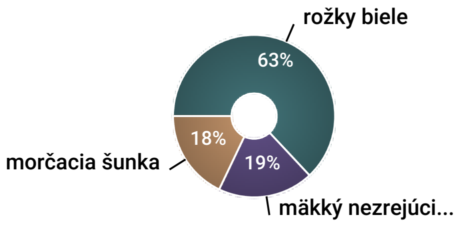

Obložené pečivo

4 min

Postup prípravy:

Pečivo obložte uvedenými ingredienciami. Ak recept uvádza aj ovocie, zjedzte ho zvlášť. Dobrú chuť.

Ďalšie ingrediencie:
ľubovoľná zelenina (ak na prísady nie ste alergická)

ENERGIA	SACHARIDY	TUKY	BIELKOVINY
263.9 kcal<br>Ideál: 222 kcal	24.97 g	9.57 g	17.69 g

INGREDIENCIE

1 x krajec chlieb pohánkový (50 g)
20 g tavený syr, 43% t. v s. napr. Apetito
2 x plátok bravčová šunka (34 g)
4 ks cherry paradajky (48 g)
1 x plátok (9×9 cm) syr eidam, 30% t. v s. neúdený (22 g)

ROZLOŽENIE ENERGIE

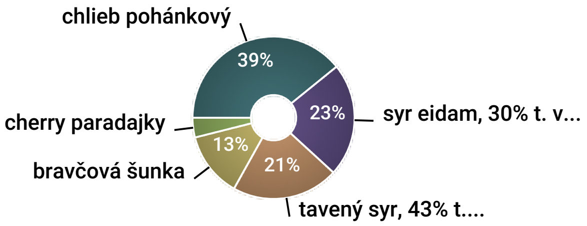

ĎALŠIE INFORMÁCIE O JEDLE

Vývar s rezancami

Postup prípravy:

Vývar konzumujte spolu s uvarenými rezancami. Dobrú chuť.

ENERGIA	SACHARIDY	TUKY	BIELKOVINY
215.86 kcal<br>Ideál: 222 kcal	37.16 g	4.12 g	6.13 g

INGREDIENCIE

35 g cestoviny dvojvaječné (neuvarené)
2 x porcia ľahký zeleninový vývar (660 g) (☰ postup prípravy nižšie)

ROZLOŽENIE ENERGIE

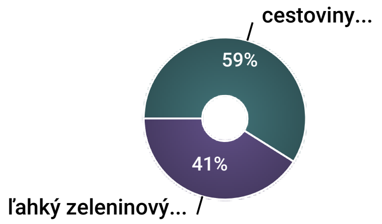

ĎALŠIE INFORMÁCIE O JEDLE

Stredná 12 GL   13 Chol

POSTUP PRÍPRAVY - ĽAHKÝ ZELENINOVÝ VÝVAR (7 x porcia = 2609g)

1 PL olivový olej (12 g)
3 x (10cm dĺžka, 10 cm obvod) pór (207 g)
1 x stredne veľká cibuľa (75 g)
3 ks mrkva (255 g)
30 x g zeler - vňať
2000 ml voda pitná (2000 g)
30 g petržlen - vňať

Postup prípravy: V polievkovom hrnci zohrejte olej. pridajte nadrobno pokrájaný pór a cibuľu, dobre premiešajte a oheň zmiernite. Prikryte natesno a 20 minút pomaly duste. Pridajte bobkový list, tymián, petržlenovú vňať, nadrobno pokrájanú mrkvu, zeler a soľ. Zalejte 2 litrami studenej vody a zosilnite oheň. Pomaly nechajte zovrieť a z povrchu počas varenia zbierajte penu a oheň znovu zmiernite. Prikryte a 35 minút pomaly varte. Vývar nalejte do veľkej ohňovzdornej nádoby a nechajte vychladnúť.

Cottage syr s pestom, zeleninou a pečivom

5 min

Postup prípravy:

Do cottage syru vmiešajte pesto. Premiešaný syr s pestom rovnomerne naneste na pečivo. Konzumujte spolu s nakrájanou zeleninou. Dobrú chuť.

Ďalšie ingrediencie:
bylinky (kurkuma, medvedí cesnak, bazalka), koreniny, pažítka (ak na prísady nie ste alergická)

ENERGIA	SACHARIDY	TUKY	BIELKOVINY
279.58 kcal<br>Ideál: 222 kcal	29.61 g	10.5 g	14.77 g

INGREDIENCIE

½ x balenie cottage cheese biely napr. Rajo (90 g)
1 x ČL pesto bazalkové BIO (9 g)
1 x krajec chlieb celozrnný ražný (ideálne kváskový) (50 g)
2 ks cherry paradajky (24 g)

ĎALŠIE INFORMÁCIE O JEDLE

14 Chol

ROZLOŽENIE ENERGIE

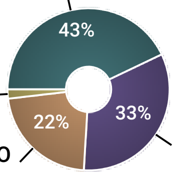

Avokádové smoothie

8 min

Postup prípravy:

Avokádo ošúpte a rozštvrťte. Potom spolu s ostatnými ingredienciami vložte do mixéra. Ak je smoothie husté, pridajte trochu vody. Dobrú chuť.

Ďalšie ingrediencie:
niekoľko lístkov 5-10 mäta, limetka zázvor podľa chuti (ak na prísady nie ste alergická)

ENERGIA	SACHARIDY	TUKY	BIELKOVINY
224.22 kcal<br>Ideál: 222 kcal	22.94 g	11.2 g	6.34 g

INGREDIENCIE

2 a ½ x čajová lyžička avokádo (37.5 g)
½ menší kus banány (32.5 g)
30 g kompót jahodový
1 a ½ dl mlieko polotučné (150 g)

ĎALŠIE INFORMÁCIE O JEDLE

7.5 Chol

ROZLOŽENIE ENERGIE

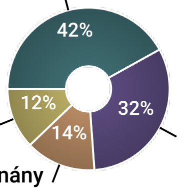

ENERGIA: **220.38 kcal** — Ideál: 222 kcal

SACHARIDY: **21.46 g**

TUKY: **11.96 g**

BIELKOVINY: **5.16 g**

INGREDIENCIE

2 a ½ x čajová lyžička avokádo (37.5 g)
75 g kompót čučoriedkový
1 a ½ dl sójové mlieko (150 g)

ĎALŠIE INFORMÁCIE O JEDLE

0 Chol

ROZLOŽENIE ENERGIE

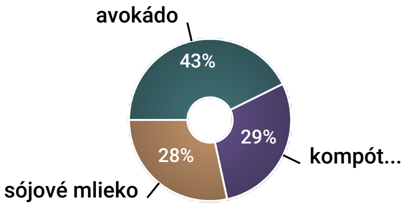

Tekvicové lečo

30 min

Postup prípravy:

Na panvici zohrejte olej. Všetku zeleninu očistite, nakrájajte na požadované kúsky a dajte restovať. Najprv cibuľku, cesnak a následne aj ostatnú zeleninu. Keď trochu zmäkne podlejte ju ešte pohárom vody. Ochuťte soľou, čiernym mletým korením, majoránkou alebo oreganom, prípadne podľa chuti môžete pridať aj chilli. Podávajte s cottage syrom a pokiaľ recept uvádza aj s pečivom alebo ryžou.

Ďalšie ingrediencie:

štipka soli, čierne mleté korenie, oregano, majoránka, chilli (ak na prísady nie ste alergická)

ENERGIA: **217.28 kcal** — Ideál: 217.56 kcal

SACHARIDY: **24.22 g**

TUKY: **6.73 g**

BIELKOVINY: **13.51 g**

INGREDIENCIE

1 a ½ strúčik cesnak (4.5 g)
60 x g tekvica
½ ks paprika červená (37.5 g)
½ ks paprika žltá (37.5 g)
½ x stredne veľká cibuľa (37.5 g)
½ ČL repkový olej rafinovaný (2.5 g)
½ x balenie cottage cheese biely napr. Rajo (90 g)
½ x krajec chlieb kukuričný (Írsky bez kvasníc a mlieka) (25 g) (☰ postup prípravy nižšie)

ĎALŠIE INFORMÁCIE O JEDLE

14 Chol

ROZLOŽENIE ENERGIE

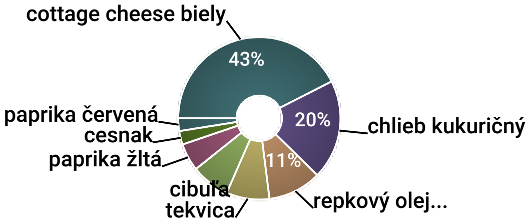

POSTUP PRÍPRAVY - CHLIEB KUKURIČNÝ (ÍRSKY BEZ KVASNÍC A MLIEKA) (19 x krajec = 965g)

450 g kukuričná múka (polenta instantná)
500 ml voda pitná (500 g)
1 x PL trstinový cukor (15 g)

Postup prípravy: Vo väčšej miske zmiešajte kukuričnú múku, soľ (1PL), cukor, sódu bikarbónu 1 ČL až PL. V strede zmesi si urobte jamku, do ktorej nalejete vodu. Jemne miešajte, kým Vám nevznikne lepivá hmota. Cesto vytvarujeme do bochníka a dajte piecť do zapekacej misy vystlanej papierom na pečenie. Ostrým nožom bochník narežte a posypte múkou. Pečte v rúre pri 180 stupňoch približne 1 hodinu.

Ovocie - mierne pásmo

Postup prípravy:

Ovocie poriadne umyte, prípadne ošúpte a pridajte k jogurtu. V prípade, že recept uvádza aj proteín, vmiešajte ho do jogurtu. Dobrú chuť.

Ďalšie ingrediencie:

podľa chuti pokvapkajte citrónovou šťavou (ak na prísady nie ste alergická)

ENERGIA	SACHARIDY	TUKY	BIELKOVINY
215.49 kcal<br>Ideál: 217.56 kcal	14.21 g	13.74 g	7.17 g

INGREDIENCIE

2 PL maliny (42 g)

1 balenie grécky jogurt napr. Bakoma (180 g)

ĎALŠIE INFORMÁCIE O JEDLE

4.3 Nízka GL   40 Chol

ROZLOŽENIE ENERGIE

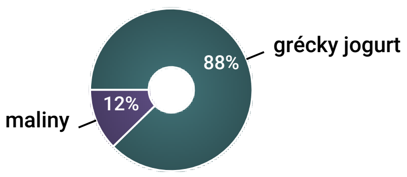

ENERGIA	SACHARIDY	TUKY	BIELKOVINY
214.24 kcal<br>Ideál: 217.56 kcal	25.41 g	9.54 g	5.22 g

INGREDIENCIE

2 a ½ x malý kus hrušky (162.5 g)

½ balenie grécky jogurt napr. Mevgal (90 g)

ĎALŠIE INFORMÁCIE O JEDLE

9.3 Nízka GL   20 Chol

ROZLOŽENIE ENERGIE

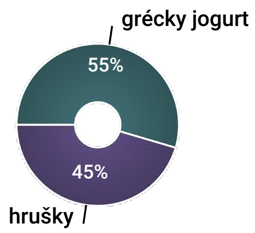

ENERGIA	SACHARIDY	TUKY	BIELKOVINY
214.59 kcal<br>Ideál: 217.56 kcal	27.43 g	7.03 g	8.96 g

INGREDIENCIE

6 PL maliny (126 g)

1 a ½ balenie jogurt biely delaktózovaný napr. Tesco (225 g)

ĎALŠIE INFORMÁCIE O JEDLE

ROZLOŽENIE ENERGIE

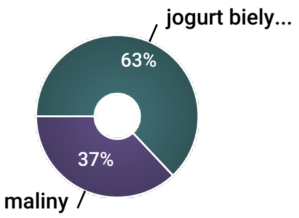

ENERGIA	SACHARIDY	TUKY	BIELKOVINY
226.42 kcal<br>Ideál: 217.56 kcal	28.08 g	9.3 g	6.04 g

INGREDIENCIE

1 a ½ ks broskyne (202.5 g)
½ balenie grécky jogurt napr. Mevgal (90 g)

ĎALŠIE INFORMÁCIE O JEDLE

11 Stredná GL
20 Chol

ROZLOŽENIE ENERGIE

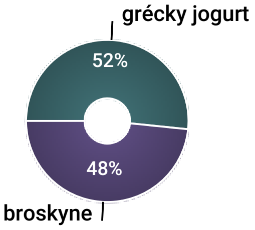

Banánový nápoj

5 min

Postup prípravy:

Všetko spolu rozmixujte a nápoj vypite. Dobrú chuť.

Ďalšie ingrediencie:
stévia (ak na prísady nie ste alergická)

ENERGIA	SACHARIDY	TUKY	BIELKOVINY
228.92 kcal<br>Ideál: 222 kcal	28.74 g	7.13 g	10.92 g

INGREDIENCIE

3 dl mlieko polotučné (300 g)
½ x čajová lyžička ľanový olej lisovaný za studena napr. Biolienka (2.5 g)
1 menší kus banány (65 g)

ĎALŠIE INFORMÁCIE O JEDLE

13 Stredná GL
15 Chol

ROZLOŽENIE ENERGIE

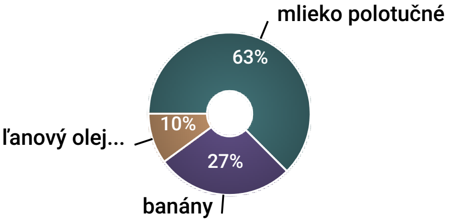

Syr so zeleninou

5 min

Postup prípravy:

Ingrediencie si pripravte na tanier a postupne konzumujte. Dobrú chuť.

Ďalšie ingrediencie:

balzamiko - vínny ocot (ak máte radi), bylinky, korenie, kari, vňať, medvedí cesnak (ak na prísady nie ste alergická)

ENERGIA	SACHARIDY	TUKY	BIELKOVINY
223.67 kcal<br>Ideál: 222 kcal	13.68 g	9.87 g	18.48 g

INGREDIENCIE

8 ks reďkovka (136 g)
½ x balenie cottage cheese biely napr. Rajo (90 g)
1 ks paprika žltá (75 g)
1 x plátok (9×9 cm) syr eidam, 45% t. v s. neúdený (22 g)

ROZLOŽENIE ENERGIE

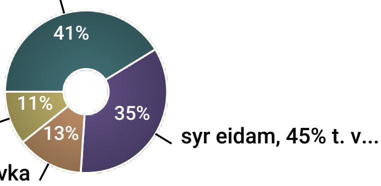

ĎALŠIE INFORMÁCIE O JEDLE

Nízka 2.1 GL 33 Chol

ENERGIA	SACHARIDY	TUKY	BIELKOVINY
219.82 kcal<br>Ideál: 222 kcal	15.2 g	7.85 g	20.6 g

INGREDIENCIE

2 ks rajčiny (150 g)
½ ks uhorky (150 g)
½ balenie cottage cheese biely napr. Tesco (90 g)
1 ks paprika červená (75 g)
1 x plátok (9×9 cm) syr eidam, 30% t. v s. neúdený (22 g)

ROZLOŽENIE ENERGIE

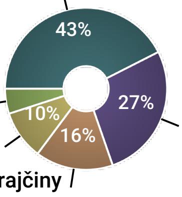

ĎALŠIE INFORMÁCIE O JEDLE

Nízka 2.1 GL 25 Chol

Večera

Červená šošovica s pečenou cviklou a syrom

30 min

Postup prípravy:

Šošovicu uvarte domäkka. Nakrájajte na kolieska červenú repu (cviklu) a upečte v rúre. Zmiešajte so šošovicou, pridajte strúhaný petržlen, cesnak, pór a olej. Pridajte syr.

Ďalšie ingrediencie:

kôpor, majoránka, štipka soli (ak na prísady nie ste alergická)

ENERGIA	SACHARIDY	TUKY	BIELKOVINY
448.55 kcal	44.29 g	15.28 g	30.45 g
Ideál: 455.9 kcal	Ideál: 44.48 g	Ideál: 14.71 g	Ideál: 33.36 g

INGREDIENCIE

1 strúčik cesnak (3 g)
1 x stredne veľký petržlen (60 g)
½ x (10cm dĺžka, 10 cm obvod) pór (34.5 g)
50 g šošovica červená napr.Country life
1 a ½ x malý kus červená repa (cvikla) (75 g)
1 a ½ ČL olivový olej (7.5 g)
½ x balenie cottage cheese (90 g)
10 g parmezán

ROZLOŽENIE ENERGIE

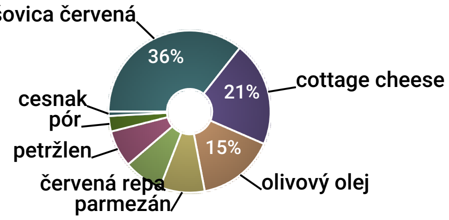

ĎALŠIE INFORMÁCIE O JEDLE

20 Chol

Tekvicové lečo

30 min

Postup prípravy:

Na panvici zohrejte olej. Všetku zeleninu očistite, nakrájajte na požadované kúsky a dajte restovať. Najprv cibuľku, cesnak a následne aj ostatnú zeleninu. Keď trochu zmäkne podlejte ju ešte pohárom vody. Ochuťte soľou, čiernym mletým korením, majoránkou alebo oreganom, prípadne podľa chuti môžete pridať aj chilli. Podávajte s vajíčkom uvareným na tvrdo (a/alebo cottage syrom) a pokiaľ recept uvádza aj s pečivom alebo ryžou.

Ďalšie ingrediencie:

štipka soli, čierne mleté korenie, oregano, majoránka, chilli (ak na prísady nie ste alergická)

ENERGIA	SACHARIDY	TUKY	BIELKOVINY
450.68 kcal	44.17 g	18.09 g	24.72 g
Ideál: 451.29 kcal	Ideál: 44.03 g	Ideál: 14.56 g	Ideál: 33.02 g

INGREDIENCIE

- *1 a ½ strúčik cesnak (4.5 g)*
- *½ x priemerný kus tekvica (125 g)*
- *½ ks paprika červená (37.5 g)*
- *½ ks paprika žltá (37.5 g)*
- *½ x (10cm dĺžka, 10 cm obvod) pór (34.5 g)*
- *1 a ½ ČL olivový olej (7.5 g)*
- *½ balenie cottage cheese biely napr. Tesco(90 g)*
- *1 x krajec chlieb zemiakový (50 g)*
- *1 x ks vajce na tvrdo (55 g)*

ROZLOŽENIE ENERGIE

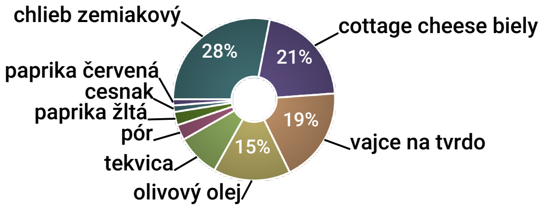

ĎALŠIE INFORMÁCIE O JEDLE

247 Chol

Sladko-pikantné karí z červenej šošovice s kuracím mäsom

🕒 45 min

Postup prípravy:

Cibuľu nakrájajte na malé kúsky a jemne opražte na oleji. Pridajte k nej nakrájané kuracie mäso a chvíľu opražte. Ďalej pridajte mrazenú zeleninu, okoreňte kari korením, zázvorom a čili (podľa chuti). Opražte 2 minúty. Pridaje opláchnutú šošovicu a zalejte vodou. Ak je zmes veľmi hustá, môžeme pridať viac vody. Varte 30 minút, alebo kým šošovica nezmäkne. Nakoniec pridajte kokosové mlieko a pasterizovaný med (len ak káže recept), kurkumu, osoľte a ešte chvíľku nechajte na miernom ohni variť. Podávajte s uvedenou prílohou.

Ďalšie ingrediencie:
soľ (ak na prísady nie ste alergická)

ENERGIA	SACHARIDY	TUKY	BIELKOVINY
466.25 kcal	47.59 g	14.71 g	32.77 g
Ideál: 455.9 kcal	Ideál: 44.48 g	Ideál: 14.71 g	Ideál: 33.36 g

INGREDIENCIE

20 x g červená cibuľa
30 g mrazená zeleninová zmes jarná
30 g šošovica červená napr.Country life
2 a ½ ČL olivový olej (12.5 g)
2 g čili korenie
2 g korenie kari
2 g kurkuma
2 g ďumbier (zázvor)
30 x g indiánska ryža (nevarená) napr. LAGRIS
65 g kuracie prsia bez kosti

ROZLOŽENIE ENERGIE

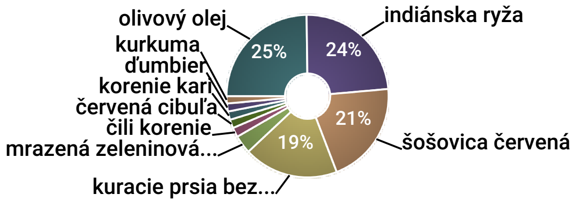

ĎALŠIE INFORMÁCIE O JEDLE

Cottage syr s pestom, zeleninou prípadne pečivom

🕒 5 min

Postup prípravy:

Do cottage syru vmiešajte pesto. Premiešaný syr s pestom rovnomerne naneste na pečivo. Konzumujte spolu s nakrájanou zeleninou. Dobrú chuť.

Ďalšie ingrediencie:
bylinky (kurkuma, medvedí cesnak, bazalka), koreniny, pažítka (ak na prísady nie ste alergická)

ENERGIA	SACHARIDY	TUKY	BIELKOVINY
460.31 kcal	45.45 g	15.94 g	30.65 g
Ideál: 460.5 kcal	Ideál: 44.93 g	Ideál: 14.85 g	Ideál: 33.7 g

INGREDIENCIE

1 x balenie cottage cheese (180 g)
1 x ČL pesto bazalkové BIO (9 g)
1 a ½ x krajec chlieb celozrnný pšeničný (ideálne kváskový) (75 g)
4 ks reďkovka (68 g)
4 ks cherry paradajky (48 g)

ROZLOŽENIE ENERGIE

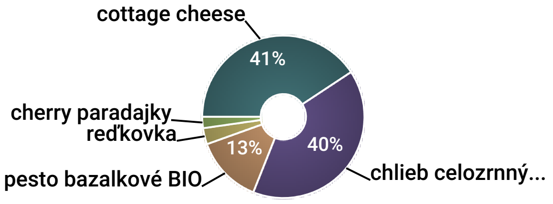

ĎALŠIE INFORMÁCIE O JEDLE

27 Chol

Cestoviny s tofu a zeleninou

🕒 25 min

Postup prípravy:

Tofu nakrájajte na kocky, pokropte sójovou omáčkou (ak na ňu nie ste alergický/á) a nechajte asi 10 min odstáť. Medzitým si na na oleji speňte nakrájanú cibuľu, rozotretý cesnak (iba ak sú v recepte uvedené). Zeleninu umyte, očistite a nastrúhajte na hrubšie pásiky. Zeleninu spolu s nakrájaným tofu pridajte na panvicu, premiešajte a duste asi 7 min za občasného miešania. Pridajte uvarené cestoviny. Dobrú chuť.

Ďalšie ingrediencie:

sójová omáčka, soľ, mleté čierne korenie, (ak na prísady nie ste alergická)

ENERGIA	SACHARIDY	TUKY	BIELKOVINY
461.99 kcal	44.81 g	14.99 g	33.87 g
Ideál: 460.5 kcal	Ideál: 44.93 g	Ideál: 14.85 g	Ideál: 33.7 g

INGREDIENCIE

105 g tofu biele napr. Alfa Bio
1 strúčik cesnak (3 g)
½ x stredne veľká cibuľa (37.5 g)
1 ČL olivový olej (5 g)
2 x väčšia naberačka cestoviny, makaróny, špagety celozrnné (varené) (120 g)
½ ks mrkva (42.5 g)
1 x porcia hovädzí vývar (bez rezancov) (330 g) (☰ postup prípravy nižšie)

ROZLOŽENIE ENERGIE

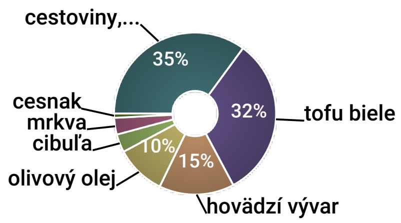

ĎALŠIE INFORMÁCIE O JEDLE

15 Stredná GL

POSTUP PRÍPRAVY - HOVÄDZÍ VÝVAR (BEZ REZANCOV) (6 x porcia = 2010g)

1 ks mrkva (85 g)
½ x priemerný kus zeler bulvový (25 g)
1 x stredne veľká cibuľa (75 g)
20 g hovädzia pečeň
4 x kus kel ružičkový (44 g)
1 strúčik cesnak (3 g)
38 g paprika zeleninová
1500 ml voda (1500 g)
2 x kocka hovädzí bujón napr. Knorr (20 g)
200 g hovädzie chudé

Postup prípravy: soľ
celé čierne korenie
100 g hovädzích kostí

Kosti dobre umyte, posekájte na menšie kúsky a vložte do studenej vody. Pridajte očistené a umyté mäso a dajte variť. Keď vývar začne vrieť, pridajte soľ, korenie a ďalej varte na veľmi miernom ohni dve hodiny. Počas varenia sa vytvára pena, ktorú môžete odstraňovať.
Po hodine varu pridajte očistenú zeleninu. Z mrkvy kúsok odložte a ten spolu s pečeňou opražte dozlatista. Vývar tak získa výraznejšiu chuť a intenzívne jantárové sfarbenie. Počas varenia z vývaru odstraňujte tuk, pretože dlhým varením dodáva nepríjemnú lojovitú príchuť a mútny odtieň.
Hotový vývar precedte cez jemné sitko. Mäso použite do polievky.
Hotovú polievku môžete dochutiť štipkou mletej červenej papriky.

Vytvorené pomocou aplikácie planeat

Cviklové rizoto

⏱ 45 min

Postup prípravy:

Nadrobno nakrájaný zeler a cibuľu poduste na tuku. Ochuťte štipkou soli, čiernym mletým korením a bobkovým listom. Keď zelenina trochu zmäkne, primiešajte neumytú ryžu. Do rizota postupne po nabračkách pridávajte vývar, vždy aby bola ryža zaliata. Po chvíli primiešajte očistenú a nastrúhaná cviklu. Asi po 20 minútach (keď je ryža uvarená) pridajte trochu citrónovej šťavy a strúhaný parmezán (len ak ho recept uvádza). Zľahka premiešajte a nechajte odpočívať. Ak by ste mali málo vývaru a ryža by stále nebola uvarená, podlejte vodou. Na tanier naservírujte pripravené rizoto, pridajte kúsky mozarelly a sezamovými semenami (len ak ich máte v recepte uvedené). Dobrú chuť.

Ďalšie ingrediencie:
soľ, čierne mleté korenie, bobkový list, citrónová šťava, (ak na prísady nie ste alergická)

ENERGIA	SACHARIDY	TUKY	BIELKOVINY
446.9 kcal<br>Ideál: 446.69 kcal	43.6 g<br>Ideál: 43.58 g	17.76 g<br>Ideál: 14.41 g	25.13 g<br>Ideál: 32.68 g

INGREDIENCIE

- *½ x stonka zeler stonkový (16.5 g)*
- *½ x stredne veľká cibuľa (37.5 g)*
- *40 g ryža (neuvarená) Basmati*
- *1 malý kus červená repa (cvikla) (50 g)*
- *1 ČL repkový olej rafinovaný (5 g)*
- *5 a ½ plátok (20g) mozzarela light napr. GALBANI (110 g)*
- *½ x hrnček ľahký zeleninový vývar (125 g) (☰ postup prípravy nižšie)*

ROZLOŽENIE ENERGIE

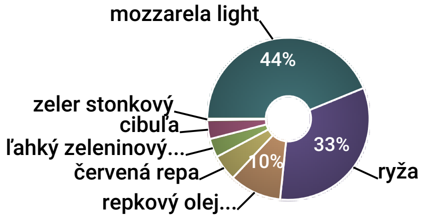

ĎALŠIE INFORMÁCIE O JEDLE

ENERGIA	SACHARIDY	TUKY	BIELKOVINY
446.8 kcal<br>Ideál: 446.69 kcal	43.6 g<br>Ideál: 43.58 g	17.75 g<br>Ideál: 14.41 g	25.12 g<br>Ideál: 32.68 g

INGREDIENCIE

- *½ x stonka zeler stonkový (16.5 g)*
- *½ x stredne veľká cibuľa (37.5 g)*
- *40 g ryža (neuvarená) Basmati*
- *1 malý kus červená repa (cvikla) (50 g)*
- *1 ČL olivový olej (5 g)*
- *5 a ½ plátok (20g) mozzarela light napr. GALBANI (110 g)*
- *½ x hrnček ľahký zeleninový vývar (125 g) (☰ postup prípravy nižšie)*

ROZLOŽENIE ENERGIE

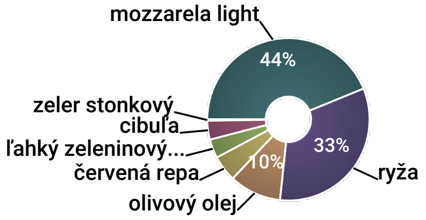

ĎALŠIE INFORMÁCIE O JEDLE

POSTUP PRÍPRAVY - ĽAHKÝ ZELENINOVÝ VÝVAR (10 x hrnček = 2609g)

- *1 PL olivový olej (12 g)*
- *3 x (10cm dĺžka, 10 cm obvod) pór (207 g)*
- *1 x stredne veľká cibuľa (75 g)*
- *3 ks mrkva (255 g)*
- *30 x g zeler - vňať*
- *2000 ml voda pitná (2000 g)*
- *30 g petržlen - vňať*

Postup prípravy: V polievkovom hrnci zohrejte olej. pridajte nadrobno pokrájaný pór a cibuľu, dobre premiešajte a oheň zmiernite. Prikryte natesno a 20 minút pomaly duste. Pridajte bobkový list, tymián, petržlenovú vňať, nadrobno pokrájanú mrkvu, zeler a soľ. Zalejte 2 litrami studenej vody a zosilnite oheň. Pomaly nechajte zovrieť a z povrchu počas varenia zbierajte penu a oheň znovu zmiernite. Prikryte a 35 minút pomaly varte. Vývar nalejte do veľkej ohňovzdornej nádoby a nechajte vychladnúť.

Nátierka z tofu a sušených paradajok

10 min

Postup prípravy:

Na panvici pokvapkanej olejom opražte nakrájené tofu (približne 5 minút). Nechajte trochu vychladnúť a dajte ju do mixéra spolu so sušenými paradajkami/pestom, pridajte horčicu a jemne osoľte. Podávajte s pečivom a zeleninou.

ENERGIA	SACHARIDY	TUKY	BIELKOVINY
461.05 kcal<br>Ideál: 460.5 kcal	45.31 g<br>Ideál: 44.93 g	16.08 g<br>Ideál: 14.85 g	30.67 g<br>Ideál: 33.7 g

INGREDIENCIE

135 g tofu biele napr. Alfa Bio
1 ČL horčica plnotučná napr. Snico (9 g)
1 ČL repkový olej rafinovaný (5 g)
1 a ½ x krajec chlieb zemiakový (75 g)
3 ks cherry paradajky (36 g)
1 ČL sušené paradajky (mleté) napr. Carthage garden (10 g)

ROZLOŽENIE ENERGIE

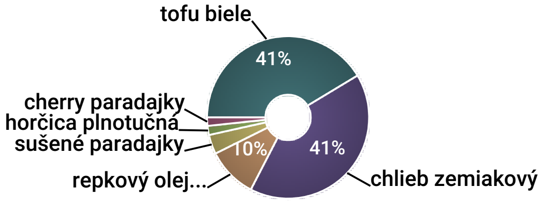

ĎALŠIE INFORMÁCIE O JEDLE

ENERGIA	SACHARIDY	TUKY	BIELKOVINY
460.96 kcal<br>Ideál: 460.5 kcal	45.32 g<br>Ideál: 44.93 g	16.07 g<br>Ideál: 14.85 g	30.67 g<br>Ideál: 33.7 g

INGREDIENCIE

135 g tofu biele napr. Alfa Bio
1 ČL horčica plnotučná napr. Snico (9 g)
1 ČL olivový olej (5 g)
1 a ½ x krajec chlieb zemiakový (75 g)
3 ks cherry paradajky (36 g)
1 ČL sušené paradajky (mleté) napr. Carthage garden (10 g)

ROZLOŽENIE ENERGIE

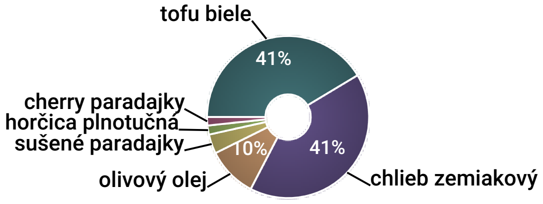

ĎALŠIE INFORMÁCIE O JEDLE

Plané mäso s prílohou a so zeleninou

20 min

Postup prípravy:

Okorenené kuracie/morčacie prsia opečte na oleji (masti, masle). Podľa druhu zeleniny ju buď orestujte na panvici, alebo zohrejte v rúre (max. 180 °C). Ľubovoľne dochuťte. Konzumujte spolu s prílohou. Dobrú chuť.

Ďalšie ingrediencie:
zeleninová soľ, korenie, bylinky (ak na prísady nie ste alergická)

ENERGIA	SACHARIDY	TUKY	BIELKOVINY
450.18 kcal	43.93 g	14.49 g	33 g
Ideál: 451.29 kcal	Ideál: 44.03 g	Ideál: 14.56 g	Ideál: 33.02 g

INGREDIENCIE

90 g morčacie prsia bez kosti
2 x menšia naberačka kuskus (varený) (100 g)
1 ks rajčiny (75 g)
½ ks uhorky (150 g)
1 a ½ ČL repkový olej rafinovaný (7.5 g)
1 x porcia brokolicová polievka (330 g) (☰ postup prípravy nižšie)

ROZLOŽENIE ENERGIE

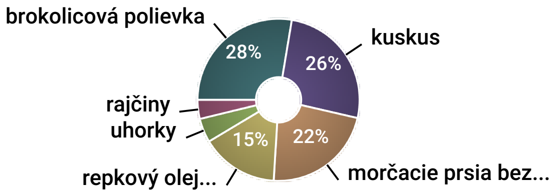

ĎALŠIE INFORMÁCIE O JEDLE

56 Chol

POSTUP PRÍPRAVY - BROKOLICOVÁ POLIEVKA (4 x porcia = 1377g)

1 x 1 balenie brokolica (500 g)
1 x stredný kus zemiaky neskoré (90 g)
1 x stredne veľká cibuľa (75 g)
2 dl mlieko polotučné 1,5% (200 g)
1 PL olivový olej (12 g)
500 ml voda pitná (500 g)

Postup prípravy: Cibuľu nakrájajte najemno a opražte ju na oleji v hrnci. Pridajte umytú, naružičkovanú brokolicu, na kocky nakrájaný zemiak a zalejte vodou tak, aby zelenina plávala. Osoľte, okoreňte a nechajte variť. Zhruba po 15 minútach, keď brokolica a zemiaky dostatočne zmäkli pridajte mlieko. Poriadne zamiešajte. Tyčovým mixérom rozmixujte celú zmes, podľa hustoty pridajte ešte mlieko. Opäť vráťte na oheň a priveďte k varu.

Pomocné ingrediencie: štipka soli, (štipka čierneho korenia) jedine ak máte odskúšané. Korenie najmä čierne vo väčšom množstve môže spôsobovať problémy

Krémová fazuľová polievka

🕒 100 min

Postup prípravy:

Fazuľu deň vopred namočte. Pred prípravou zlejte a zalejte čerstvou vodou a dajte variť. Pridajte nakrájaný petržlen a mrkvu. Všetko spolu nechajte variť na miernom ohni, až kým fazuľa dostatočne nezmäkne. Keď máte fazuľu uvarenú, naberačkou odoberte do taniera asi polovicu fazule a čo najviac mrkvy. Zvyšnú fazuľu a petržlen spolu s natrhanou mozzarelou (ak je uvedená v recepte) v hrnci rozmixujte ponorným mixérom na hladkú konzistenciu. Pridajte naspäť zvyšnú fazuľu a mrkvu, dochuťte čiernym korením, soľou, pridajte olej a pretlačený cesnak. Všetko zamiešajte a nechajte krátko prevariť. Pre viac porcií len roznásobte ingrediencie.

Ďalšie ingrediencie:
štipka soli, čierne korenie (ak na prísady nie ste alergická)

ENERGIA	SACHARIDY	TUKY	BIELKOVINY
455.31 kcal	49.18 g	16.02 g	25.54 g
Ideál: 455.9 kcal	Ideál: 44.48 g	Ideál: 14.71 g	Ideál: 33.36 g

INGREDIENCIE

70 x g fazuľa
½ strúčik cesnak (1.5 g)
½ ks mrkva (42.5 g)
½ x stredne veľký petržlen (30 g)
1 ČL olivový olej (5 g)
45 g mozarela

ROZLOŽENIE ENERGIE

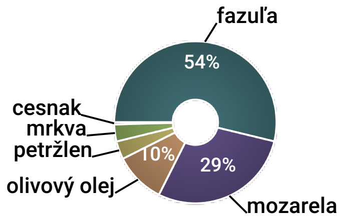

ĎALŠIE INFORMÁCIE O JEDLE

Losos so zeleninou na večeru

🕒 20 min

Postup prípravy:

Lososa umyte pod tečúcou vodou. Narežte doňho tenké rezy (nekrájajte ho) a vložte do nich plátky rajčiny a citrónu. Rybu zabaľte do alobalu a nechajte vo vopred ohriatej rúre piecť 13-18 minút pri 180 °C. Konzumujte spolu s podusenou zeleninou, alebo zemiakmi (ako káže recept). Dochuťte troškou zeleninovej soli.

Ďalšie ingrediencie:
zeleninová soľ (ak na prísady nie ste alergická)

ENERGIA	SACHARIDY	TUKY	BIELKOVINY
458.94 kcal	45.13 g	15.13 g	32.48 g
Ideál: 455.9 kcal	Ideál: 44.48 g	Ideál: 14.71 g	Ideál: 33.36 g

INGREDIENCIE

135 g losos obyčajný
2 a ½ ks rajčiny (187.5 g)
½ x kus citróny (60 g)
2 a ½ x väčšia naberačka zemiaky nové varené (150 g)

ROZLOŽENIE ENERGIE

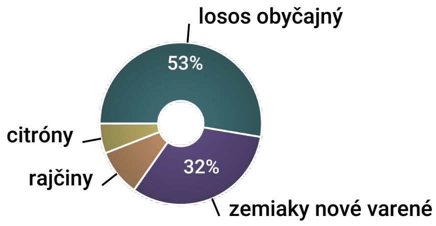

ĎALŠIE INFORMÁCIE O JEDLE

22 Vysoká GL   72 Chol

ENERGIA	SACHARIDY	TUKY	BIELKOVINY
456.37 kcal<br>Ideál: 455.9 kcal	44.44 g<br>Ideál: 44.48 g	14.79 g<br>Ideál: 14.71 g	33.32 g<br>Ideál: 33.36 g

INGREDIENCIE

130 g losos obyčajný
1 miska (400ml) zelené fazuľové struky (mrazené) napr. Bonduelle (160 g)
1 a ½ ks rajčiny (112.5 g)
½ x kus citróny (60 g)
2 x väčšia naberačka zemiaky neskoré varené (120 g)

ROZLOŽENIE ENERGIE

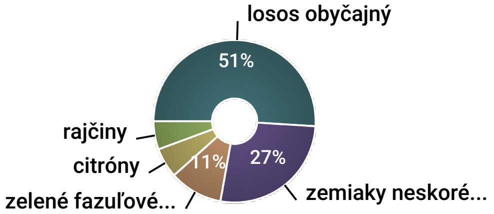

ĎALŠIE INFORMÁCIE O JEDLE

18 Stredná GL   69 Chol

ENERGIA	SACHARIDY	TUKY	BIELKOVINY
456.91 kcal<br>Ideál: 455.9 kcal	45.02 g<br>Ideál: 44.48 g	14.59 g<br>Ideál: 14.71 g	33.33 g<br>Ideál: 33.36 g

INGREDIENCIE

130 g losos obyčajný
1 a ½ x miska 300 ml zeleninová zmes mrazená (kukurica, hrach, mrkva, karfiol) napr. Minestrone, Bonduelle (232.5 g)
1 ks rajčiny (75 g)
½ x kus citróny (60 g)
1 x väčšia naberačka zemiaky neskoré varené (60 g)

ROZLOŽENIE ENERGIE

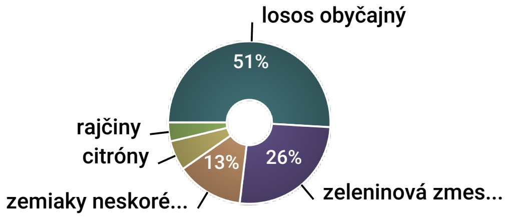

ĎALŠIE INFORMÁCIE O JEDLE

9.4 Nízka GL   69 Chol

Hovädzie plátky s pórom a prílohou

60 min

Postup prípravy:

Mäso umyte, osušte, osoľte. Opečte na oleji (masle, masti) zo všetkých strán. Podlejte vodou a duste pod pokrievkou. Medzitým očistite a umyte pór. Po dĺžke ho rozrežte a nakrájajte na prúžky. Na panvici ho poduste, osoľte, okoreňte. Mäkké mäso nakrájajte na plátky, na tanieri obložte poduseným pórom. Konzumujte s prílohou.

Ďalšie ingrediencie:

soľ, korenie, bylinky (ak na prísady nie ste alergická)

ENERGIA	SACHARIDY	TUKY	BIELKOVINY
451.07 kcal	43.96 g	14.58 g	32.98 g
Ideál: 455.9 kcal	Ideál: 44.48 g	Ideál: 14.71 g	Ideál: 33.36 g

INGREDIENCIE

115 g hovädzia sviečkovica
1 x ČL živočíšne maslo (5 g)
½ x (10cm dĺžka, 10 cm obvod) pór (34.5 g)
2 x väčšia naberačka tarhoňa (varená) (120 g)
1 x porcia polievka zeleninová- jarná (bez rezancov a halušiek) (330 g) (☰ postup prípravy nižšie)

ROZLOŽENIE ENERGIE

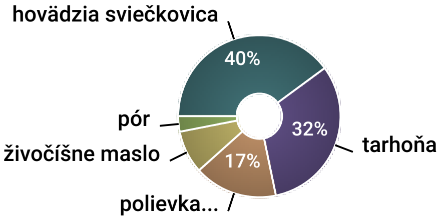

ĎALŠIE INFORMÁCIE O JEDLE

89 Chol

POSTUP PRÍPRAVY - POLIEVKA ZELENINOVÁ- JARNÁ (BEZ REZANCOV A HALUŠIEK) (9 x porcia = 3090g)

4 x väčšia naberačka zemiaky neskoré (240 g)
2 ks mrkva (170 g)
1 x stredne veľký petržlen (60 g)
1 x stredne veľká cibuľa (75 g)
1 strúčik cesnak (3 g)
200 g brokolica mrazená napr.(Bonduelle)
100 g karfiol
230 g hrach mrazený napr. Vitastar
1 PL olivový olej (12 g)
2000 ml voda (2000 g)

Postup prípravy: 35 minút

vegeta bez glutamanu sodného, korenie čierne drvené (iba ak tolerujete), paprika
červená mletá sladká, zelerová vňať, petržlenová vňať

1. Zeleninu očistite a najkrájajte nadrobno
2. Do hrnca nalejte olej a osmažte cibuľu. Ak je zlatistá pridajte nakrájanú zeleninu spolu s nakrájanými zemiakmi a posmažte na oleji. Na konci pridajte cesnak, soľ a koreniny a prilejte vodu
3. Keď začne voda vrieť, pridajte zelerovú a petržlenovú vňať a povarte asi 10 min.
4. Keď je zelenina skoro uvarená pridajte karfiol a brokolicu. Varte ďalej, až kým bude zelenina mäkká. Ak je mäkká pridajte na záver mrazený hrach. Ľubovoľne dochuťte koreninami

Rizoto s kuracím mäsom a zeleninou

🕒 60 min

Postup prípravy:

Ryžu uvarte v osolenej vode domäkka (približne 15 minút, podľa typu ryže). Uvarenú ryžu prepláchnite vlažnou vodou a nechajte odkvapkať. Očistenú mrkvu a stonkový zeler nakrájajte na malé kocky. Zeleninu opečte na rozohriatom tuku. Dochuťte štipkou soli, čiernym mletým korením. Keď zelenina trochu zmäkne, pridajte nakrájané šampiňóny a premiešajte. Chvíľu opekajte a pridajte na polovice nakrájané paradajky. Nakoniec pridajte uvarenú ryžu a umytý špenát. Zľahka premiešajte kým sa špenát sparí. Podávajte s pečeným kurčaťom. Dobrú chuť.

Ďalšie ingrediencie:
soľ, čierne mleté korenie, chilli paprička (ak na prísady nie ste alergická)

ENERGIA	SACHARIDY	TUKY	BIELKOVINY
449.36 kcal	43.52 g	14.73 g	32.68 g
Ideál: 446.69 kcal	Ideál: 43.58 g	Ideál: 14.41 g	Ideál: 32.68 g

INGREDIENCIE

½ x stonka zeler stonkový (16.5 g)
45 g ryža (neuvarená) Basmati
½ ks mrkva (42.5 g)
3 ks šampiňóny čerstvé (78 g)
1 ČL repkový olej rafinovaný (5 g)
1 miska objem (300 ml) špenát (20 g)
3 ks cherry paradajky (36 g)
125 g Pečené kura (☰ postup prípravy nižšie)

ROZLOŽENIE ENERGIE

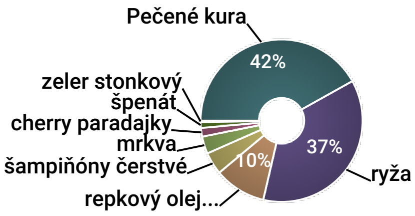

ĎALŠIE INFORMÁCIE O JEDLE

POSTUP PRÍPRAVY - PEČENÉ KURA (1012 g)

1000 g kurča domáce
1 PL olivový olej (12 g)

Postup prípravy: Umyté a osušené kurča dôkladne osoľte a okoreňte. Preložte do zapekacej misy, pokvapkajte olejom, posypte tymiánom a podlejte trochou vody. Pečte vo vyhriatej rúre pri 200 stupňoch približne 1 hodinu. Počas pečenia podlievajte kura vlastným výpekom. Upečené rozporciujte.

Basmati rizoto s pečenou špargľou a lososom

30 min

Postup prípravy:

Filety lososa umyte, osoľte a dajte do parného hrnca na 15 min alebo do vody a povarte. Nakrájajte špargľu a opražte na masle do chrumkava. Ryžu chvíľku popražte, zalejte mliekom zmiešaným s vodou a povarte domäkka. Potom pridajte parmezán a miešajte asi 3 min. Pridajte pečenú špargľu, kúsky lososa a trochu masla na chuť. Dobú chuť.

Ďalšie ingrediencie:
štipka soli (ak na prísady nie ste alergická)

ENERGIA	SACHARIDY	TUKY	BIELKOVINY
456.25 kcal	44.6 g	16.09 g	30.18 g
Ideál: 455.9 kcal	Ideál: 44.48 g	Ideál: 14.71 g	Ideál: 33.36 g

INGREDIENCIE

45 x g ryža (neuvarená) Basmati
3 x kus (25 cm dĺžka) špargľa (63 g)
100 g losos obyčajný
1 a ½ dl mlieko polotučné (150 g)
½ x čajová lyžička živočíšne maslo (3.5 g)

ROZLOŽENIE ENERGIE

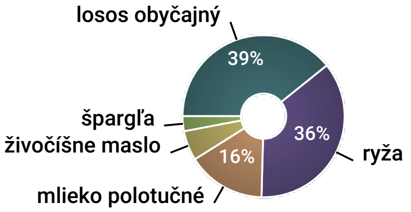

ĎALŠIE INFORMÁCIE O JEDLE

Syrová večera so zeleninou

7 min

Postup prípravy:

Ľahké jedlo v podobe cottage syra alebo mozzarelly spolu s pečivom (ak sa nachádza v recepte) a zeleninovou prílohou. Dochuťte štipkou zeleninovej soli, prípadne balzamikom (podľa chuti). Dobrú chuť.

Ďalšie ingrediencie:
zeleninová soľ, balzamiko (ak na prísady nie ste alergická)

ENERGIA	SACHARIDY	TUKY	BIELKOVINY
455.33 kcal	44.67 g	14.85 g	32.71 g
Ideál: 460.5 kcal	Ideál: 44.93 g	Ideál: 14.85 g	Ideál: 33.7 g

INGREDIENCIE

½ x balenie cottage cheese biely napr. Rajo (90 g)
6 a ½ x PL cícer varený (123.5 g)
2 ks rajčiny (150 g)
8 ks reďkovka (136 g)
3 x plátok priemer 14.5 cm morčacia šunka (54 g)
1 a ½ x čajová lyžička ľanový olej lisovaný za studena napr. Biolienka (7.5 g)

ROZLOŽENIE ENERGIE

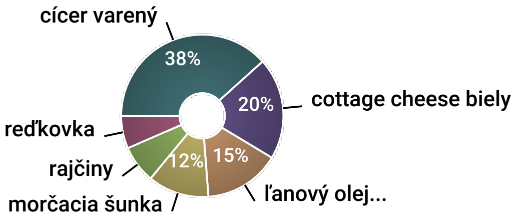

ĎALŠIE INFORMÁCIE O JEDLE

12 Stredná GL
19 Chol

Francúzske zemiaky

🕒 60 min

Postup prípravy:

Zemiaky najskôr uvarte v šupke, potom očistite a nakrájajte na tenké plátky. Na dno pekáča natrite olej.Dajte prvú vrstvu zemiakov. Na ne trochu soli a čierneho korenia. Na to ukladajte vrstvu natvrdo uvarených vajec (bielkov), nakrájaných na tenké plátky. Pridajte plátky syra alebo strúhaný syr. Následne navrch naukladajte koliečka šunky. Zalejte smotanou. Vrstvy opakujte. Zakryte alobalom a dajte do trúby na 45-60 minút. Po ukončení ich popečte ešte asi 15 minút bez alobalu. Dobrú chuť.

Ďalšie ingrediencie:

štipka soli, čierne korenie (ak na prísady nie ste alergická)

ENERGIA	SACHARIDY	TUKY	BIELKOVINY
450.24 kcal<br>Ideál: 455.9 kcal	46.06 g<br>Ideál: 44.48 g	15.09 g<br>Ideál: 14.71 g	29.51 g<br>Ideál: 33.36 g

INGREDIENCIE

- *3 a ½ x väčšia naberačka zemiaky skoré (210 g)*
- *1 a ½ ČL repkový olej rafinovaný (7.5 g)*
- *3 x veľkosť M slepačí bielok (111 g)*
- *1 x plátok (9×9 cm) Syr eidam 30 % TUKU napr. BLUEDINO LIDL (22 g)*
- *1 a ½ x plátok bravčová šunka (25.5 g)*
- *1 a ½ PL smotana kyslá pochúťková napr. Rajo (18 g)*
- *3 (8cm) ks uhorky zavárané kyslé napr. Bioline (102 g)*

ROZLOŽENIE ENERGIE

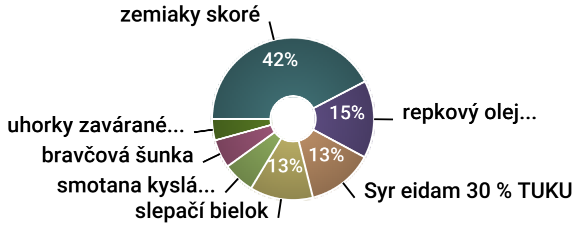

ĎALŠIE INFORMÁCIE O JEDLE

Rizoto so sušenými paradajkami

🕒 45 min

Postup prípravy:

Nadrobno nakrájanú cibuľu orestujte na tuku do sklovita. Pridajte neumytú ryžu, ktorú orestujte asi 1 minútu. Postupne podlievajte vývarom, aby bola ryža zakrytá. Varte asi 18-20 minút, nie úplne do mäkka. Ak by bolo vývaru málo, podlievajte už len vodou. Rizoto dochuťte nastrúhaným parmezánom (len ak ho recept uvádza). Rizoto by malo byť krémové. Naservírujte do hlbokého taniera, ozdobte sušenými paradajkami a cottage syrom (len ak ho recept uvádza). Dobrú chuť.

Ďalšie ingrediencie:

soľ, biele víno, bazalka (ak na prísady nie ste alergická)

ENERGIA	SACHARIDY	TUKY	BIELKOVINY
458.74 kcal<br>Ideál: 460.5 kcal	47.99 g<br>Ideál: 44.93 g	17.55 g<br>Ideál: 14.85 g	24.09 g<br>Ideál: 33.7 g

INGREDIENCIE

- *2 ČL sušené paradajky (mleté) napr. Carthage garden (20 g)*
- *½ x stredne veľká cibuľa (37.5 g)*
- *45 g ryža (neuvarená) Basmati*
- *1 ČL olivový olej (5 g)*
- *95 x g cottage cheese biely napr. Tesco*
- *½ x šálka(250ml) polievka slepačia (bez rezancov) (125 g) (☰ postup prípravy nižšie)*

ROZLOŽENIE ENERGIE

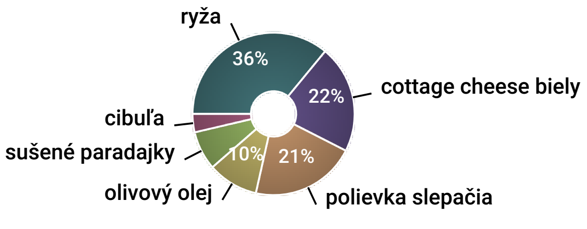

ĎALŠIE INFORMÁCIE O JEDLE

POSTUP PRÍPRAVY - POLIEVKA SLEPAČIA (BEZ REZANCOV) (19 x šálka(250ml) = 4858g)

½ x kus sliepka domáca (1500 g)
3000 ml voda (3000 g)
1 x stredne veľký petržlen (60 g)
1 x priemerný kus zeler bulvový (50 g)
1 x stredne veľká cibuľa (75 g)
1 strúčik cesnak (3 g)
2 ks mrkva (170 g)

Postup prípravy: 4 ks nové korenie
4 ks mleté čierne korenie
petržlenová vňať
soľ

Slepačie mäso očistite a spolu s kosťami vložte do studenej vody. Pridajte očistenú zeleninu a koreniny a varte do mäkka, cca 2 hodiny. Po uvarení mäkka a zeleniny, polievku precedte. Slepačie mäso a mrkvu pokrájajte na drobné kúsky a spolu s pokrájanou petržlenovou vňaťou alebo pažítkou vložte do polievky. V prípade potreby dochuťte.

Cestoviny so zeleninovou omáčkou

25 min

Postup prípravy:

Cestoviny uvarte podľa návodu na obale. Medzitým na tuku opražte nakrájanú slaninku (ak je uvedené v recepte), nadrobno nakrájanú cibuľku a cesnak. Následne pridajte paradajkové pyré a zeleninu. Nechajte povariť domäkka. Do omáčky pridajte tvaroh/lučinu, jemne osoľte a okoreňte a pridajte oregano. Omáčku podávajte spolu s uvarenými cestovinami, posypte parmezánom (ak ho máte v recepte uvedený). Dobrú chuť.

ENERGIA	SACHARIDY	TUKY	BIELKOVINY
463.48 kcal	48.66 g	14.93 g	30.52 g
Ideál: 460.5 kcal	Ideál: 44.93 g	Ideál: 14.85 g	Ideál: 33.7 g

INGREDIENCIE

1 a ½ ČL repkový olej rafinovaný (7.5 g)
1 strúčik cesnak (3 g)
½ x stredne veľká cibuľa (37.5 g)
2 x PL paradajkové pyré napr. Valfrutta (28 g)
55 g mrazený karfiol
5 a ½ PL tvaroh jemný hrudkový 2,5% napr. Pilos (93.5 g)
55 g cestoviny celozrnné, bezvaječné (neuvarené)
15 g parmezán

ROZLOŽENIE ENERGIE

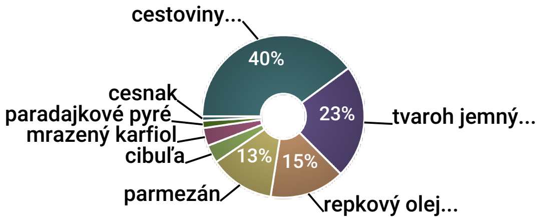

ĎALŠIE INFORMÁCIE O JEDLE

Špenátová omeleta s tuniakom

15 min

Postup prípravy:

Vajíčka rozšľahajte so soľou. Špenát opláchnite a sparte pod horúcou vodou. Nalejte na rozpálenú panvicu (jemne pokropenú olejom) a upečte z jednej strany, potom posypte sparenými listami špenátu a kúskami tuniaka. Vložte do rúry alebo prikryte pokrievkou, aby sa upiekla aj z vrchnej strany. Konzumujte s pečivom a syrom (ak je uvedený v recepte). Dobrú chuť.

Ďalšie ingrediencie:
štipka soli (ak na prísady nie ste alergická)

ENERGIA	SACHARIDY	TUKY	BIELKOVINY
451.02 kcal	42.28 g	14.87 g	34 g
Ideál: 455.9 kcal	Ideál: 44.48 g	Ideál: 14.71 g	Ideál: 33.36 g

INGREDIENCIE

1 ks slepačie vajce (55 g)
2 miska objem (300 ml) špenát (40 g)
8 x kus chlebíček BIO ryžový napr. Racio (48 g)
5 ČL mäkký nezrejúci syr napr. Lučina (30 g)
1 a ½ x malá konzerva tuniak vo vlastnej šťave napr. Rio Mare (84 g)

ROZLOŽENIE ENERGIE

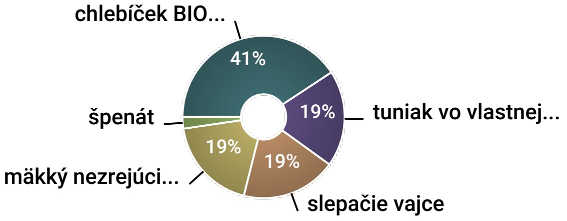

ĎALŠIE INFORMÁCIE O JEDLE

Fazuľový šalát s jogurtovým dipom

20 min

Postup prípravy:

Fazuľu namočte na 24 hodín, vložte do novej vody a povarte domäkka. V prípade fazule z konzervy ju použite bez varenia. Fazuľu zmiešajte s paradajkami a jogurtom. Cibuľu, cesnak a semienka poriadne na panvici opražte a pridajte k fazuľovej zmesi. Podávajte s nakrájaným uvareným vajíčkom.

Ďalšie ingrediencie:
štipka soli (ak na prísady nie ste alergická)

ENERGIA	SACHARIDY	TUKY	BIELKOVINY
456.79 kcal	45.06 g	14.71 g	32.99 g
Ideál: 455.9 kcal	Ideál: 44.48 g	Ideál: 14.71 g	Ideál: 33.36 g

INGREDIENCIE

60 x g fazuľa ADZUKI
½ strúčik cesnak (1.5 g)
½ x kus (40 cm) jarná cibuľka (22 g)
½ ks rajčiny (37.5 g)
½ miska objem (300 ml) rukola (8.5 g)
2 a ½ PL jogurt biely (min. 3,5% tuku) (50 g)
2 a ½ x čajová lyžička konopné semienka lúpané napr. BioCare (8.75 g)
½ x ks vajce na tvrdo (27.5 g)
1 ČL olivový olej (5 g)

ROZLOŽENIE ENERGIE

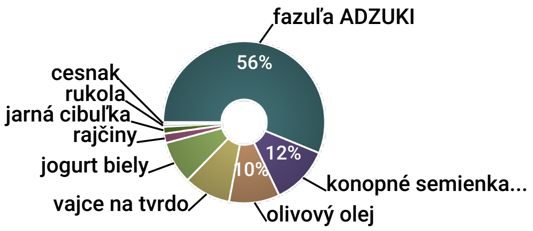

ĎALŠIE INFORMÁCIE O JEDLE

Menu - Večera

Rizoto z planého mäsa

Postup prípravy:

Rizoto ako menu variant v reštauračných zariadeniach. Tento recept si môžete spraviť aj doma, nie len ako menu variant.

Ďalšie ingrediencie:
korenie, zeleninová soľ, bylinky, huby, zelenina, tvrdý syr (posypať), zavárané uhorky (ak na prísady nie ste alergická)

ENERGIA	SACHARIDY	TUKY	BIELKOVINY
345.55 kcal	33.62 g	11.12 g	25.45 g
Ideál: 345.38 kcal	Ideál: 33.7 g	Ideál: 11.14 g	Ideál: 25.27 g

INGREDIENCIE

75 g kuracie prsia bez kosti
2 väčšia naberačka ryža varená napr. basmati, Vitana (120 g)
2 ČL repkový olej rafinovaný (10 g)

ĎALŠIE INFORMÁCIE O JEDLE

58 Chol

ROZLOŽENIE ENERGIE

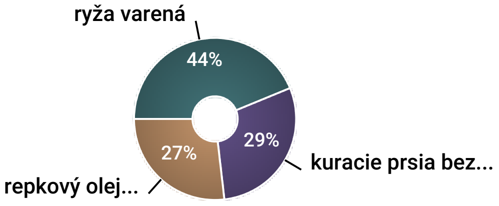

Hovädzie, teľacie mäso s prílohou

Postup prípravy:

Hovädzie (resp. teľacie ak je uvedené vo variante) mäso s prílohou plus zelenina, ako menu variant v reštauračných zariadeniach. Ak Vám ponúknu poliať mäso mastnou omáčkou, zdvorilo odmietnite :) Túto kombináciu si môžete pripraviť aj doma, nie len ako „menu variant". Dobrú chuť.

Ďalšie ingrediencie:
korenie, bylinky, soľ, zeleninová obloha (ak na prísady nie ste alergická)

ENERGIA	SACHARIDY	TUKY	BIELKOVINY
391.54 kcal	37.54 g	12.85 g	28.82 g
Ideál: 391.43 kcal	Ideál: 38.19 g	Ideál: 12.63 g	Ideál: 28.64 g

INGREDIENCIE

95 g hovädzie mäso varené
1 a ½ väčšia naberačka ryža varená napr. basmati, Vitana (90 g)
1 ČL repkový olej rafinovaný (5 g)
1 x porcia zeleninová krémová polievka (330 g) (☰ postup prípravy nižšie)

ĎALŠIE INFORMÁCIE O JEDLE

82 Chol

ROZLOŽENIE ENERGIE

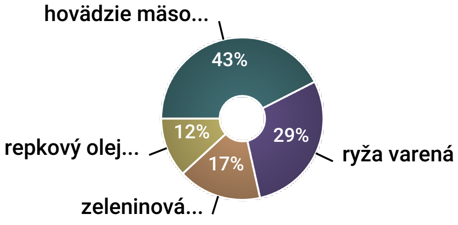

POSTUP PRÍPRAVY - ZELENINOVÁ KRÉMOVÁ POLIEVKA (6 x porcia = 2294g)

3 ks mrkva (255 g)
½ x 1 balenie brokolica (250 g)
2 x stredný kus zemiaky neskoré (180 g)
3 strúčik cesnak (9 g)
100 g hrášok
1500 ml voda pitná (1500 g)

Postup prípravy: Zeleninu očistite a nakrájajte na menšie časti. Vložte do hrnca, zalejte vodou a varte, kým zelenina nie je mäkká. Pridajte čierne korenie a soľ. Polievku vymixujte ponorným mixérom dohladka.

Pomocné ingrediencie: štipka soli, (štipka čierneho korenia) jedine ak máte odskúšané. Korenie najmä čierne vo väčšom množstve môže spôsobovať problémy
